# Video reconstruction of variable VLBI observations with neural fields

**DOI:** 10.1038/s41586-026-10988-5

**Published:** 2026-08-26

**Authors:** Marianna Foschi, Brandon Zhao, Antonio Fuentes, Katherine L. Bouman, José L. Gómez, Aviad Levis

**Affiliations:** 1 Instituto de Astrofísica de Andalucía (IAA-CSIC), Granada, Spain; 2 Division of Physics, Mathematics and Astronomy, Caltech, Pasadena, CA USA; 3 Department of Computing and Mathematical Sciences, Caltech, Pasadena, CA USA; 4 Department of Computer Science, University of Toronto, Toronto, Ontario Canada; 5 David A. Dunlap Department of Astronomy & Astrophysics, University of Toronto, Toronto, Ontario Canada

**Keywords:** Astronomy and astrophysics, Astronomy and astrophysics

## Abstract

Supermassive black hole accretion and the ejection of collimated, relativistic jets of plasma are intrinsically dynamic processes shaped by large-scale magnetic fields^1–4^. Various algorithms have been developed to image these objects at different scales using radio interferometric observations^5–8^. However, there is a lack of imaging methods that can robustly resolve the temporal variability of the sources at high resolution. Here we present kine, a video reconstruction algorithm for very long baseline interferometry observations of variable sources. The kine algorithm uses a neural representation^9^ of the video to simultaneously process observations at different times, while learning and leveraging the spatio-temporal correlations present in the data. The algorithm reconstructs polarimetric time-continuous videos from single observations of fast-varying sources, such as horizon-scale observations of Sagittarius A* with the Event Horizon Telescope, or from repeated observations of slowly varying sources. In this work, we demonstrate the latter case, applying kine to multi-epoch Very Long Baseline Array observations of blazar 3C 345 (ref. ^10^). The time continuity of the video, combined with the resolution and dynamic range improvement achieved over traditional methods, enables the measurement of the local, instantaneous velocity of the plasma in the jet, in contrast to previous methods that track only discrete components. The proposed algorithm and methodology provide a transformative tool for kinematic jet analysis and can be applied to entire monitoring programs, providing a complete kinematic description of hundreds of sources, possibly leading to a reinterpretation of established models.

## Abstract (web summary)

The kine neural reconstruction algorithm produces high-resolution, time-continuous very long baseline interferometry videos, enabling direct measurement of instantaneous plasma velocities and detailed kinematic analysis of relativistic astrophysical jets.

## Main

Supermassive black holes at the centre of active galactic nuclei drive the ejection of jets^1,2^, highly relativistic, collimated streams of plasma that propagate through the intergalactic medium^11^. Very long baseline interferometry (VLBI) is a primary tool to observe jets, enabling the detection of broad, unresolved features, called components, moving downstream at apparent superluminal speeds^12–15^. Superluminal components have been associated with travelling shock waves that compress the magnetized plasma^16^, whereas alternative models explain the components with plasma instabilities and geometrical effects^17,18^.

Traditional VLBI imaging relies on deconvolution techniques such as the widely adopted CLEAN^5^ algorithm. In recent years, alternative forward-modelling imaging approaches have been proposed^6–8^, which achieve improved angular resolution by incorporating prior assumptions about the image in a parametrized model of the sky brightness^18^.

We present a forward-modelling VLBI imaging method, kine, that recovers a full-polarimetric video of variable radio sources. kine reconstructs the video by jointly recovering all frames simultaneously, learning and enforcing the spatio-temporal correlations in the data through a neural representation of the video, which infers the extent of the correlations during the imaging process. This method outputs a continuous representation of the brightness density distribution, sampleable at any time coordinate. By imaging multiple datasets simultaneously, each frame is reconstructed using substantially more information than in traditional, single-epoch imaging, leading to improved angular resolution and dynamic range that can overcome limited (*u*, *v*)-coverage or lower signal-to-noise ratio (SNR) on individual observations.

The continuity and high-resolution of our video allow us to apply post-processing techniques, such as optical flow^19,20^, to recover the local instantaneous velocity field associated with the plasma. This is an advance over traditional kinematic analyses based on Gaussian model fitting^21,22^ or wavelet component decomposition^23^ because it resolves in space and time the projected velocity field describing the apparent motion of the emission of the source, rather than inferring motion by tracking discrete components. Assuming that spatial changes in the emission correspond to motion of the jet plasma, our method measures both the plasma bulk motion and the velocity of individual bright components, whereas previous methods could only measure the latter.

We showcase the performance of kine on 116 observations of blazar 3C 345 at 15 GHz, conducted from 1995 to 2022, by the Very Long Baseline Array (VLBA) as part of the MOJAVE monitoring program^10^. At a redshift of *z* = 0.593 and an inclination of 3°–6.8° from the line of sight^22,24^, 3C 345 has been monitored for five decades^25,26^, with recent observations showing a variable relativistic jet, characterized by the ejection of numerous superluminal components interpreted as moving shocks^21,22,24,27–29^. With our imaging method, paired with motion analysis tools, we recovered a continuous, high-resolution, high-dynamic range polarimetric video of 3C 345 and measured the two-dimensional (2D) velocity field in the jet plasma, shedding light on the nature of jet dynamics and superluminal components.

**Fig. 1:**
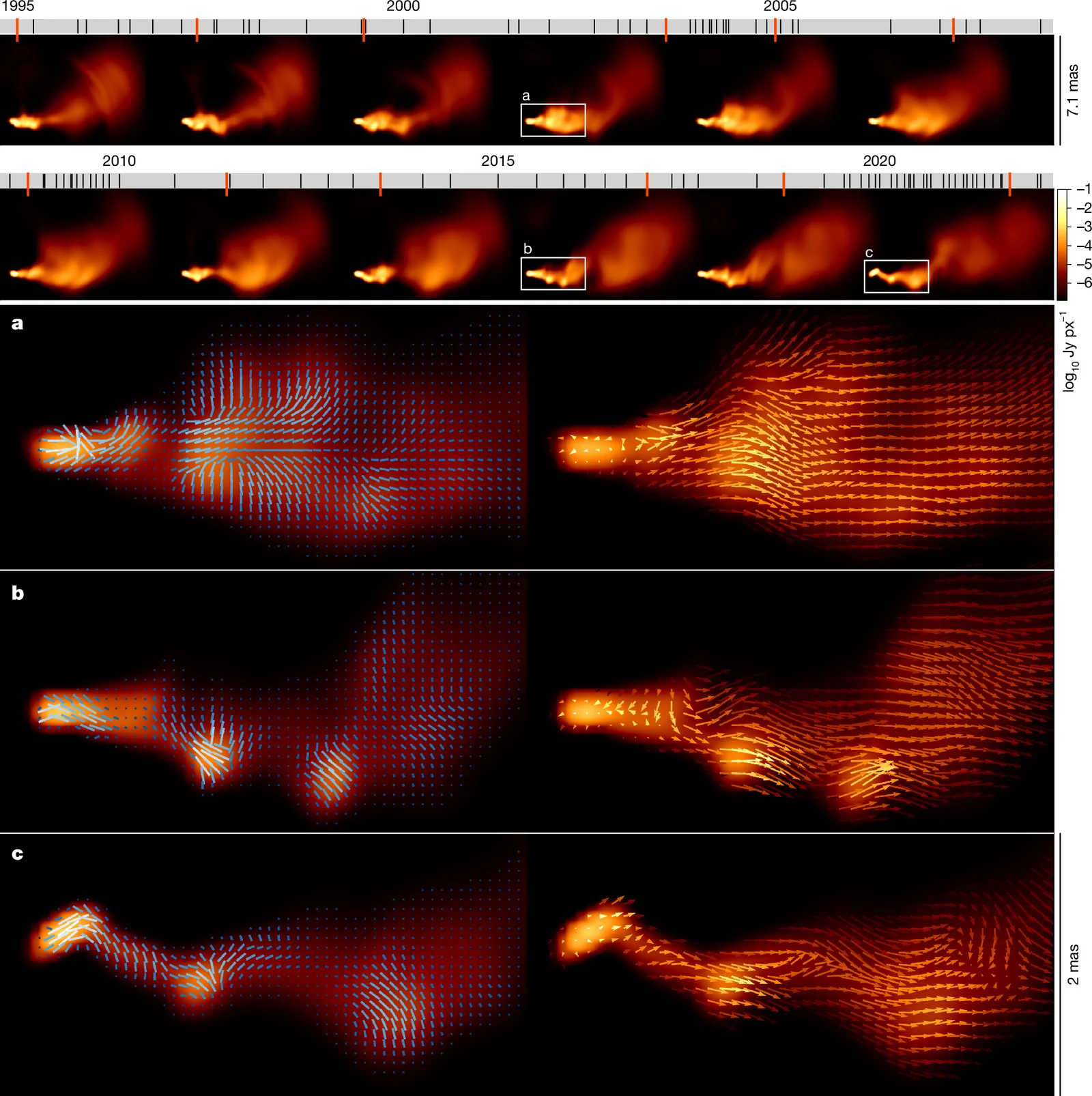
Time-resolved relativistic jet flow in 3C 345. The frames at the top show the kine video reconstruction of 116 epochs from the MOJAVE program at 15 GHz. **a**–**c**, Magnification of the inner jet, showing linear polarization (left) and velocity field (right). The linear polarization ticks are proportional to the logarithm of the polarization field, with displayed maximum values of 5.3 × 10^−3^ Jy px^−1^. The velocity field arrows are proportional to the instantaneous velocity, with displayed maximum values of 9.0 c. The colour of the arrows matches the colour of the underlying total intensity image. The dates corresponding to the selected frames are marked red on the timelines. The *x*- and *y*-axes of the frames correspond to right ascension (RA) and declination (Dec) coordinates, respectively.

**Extended Data Fig. 1:**
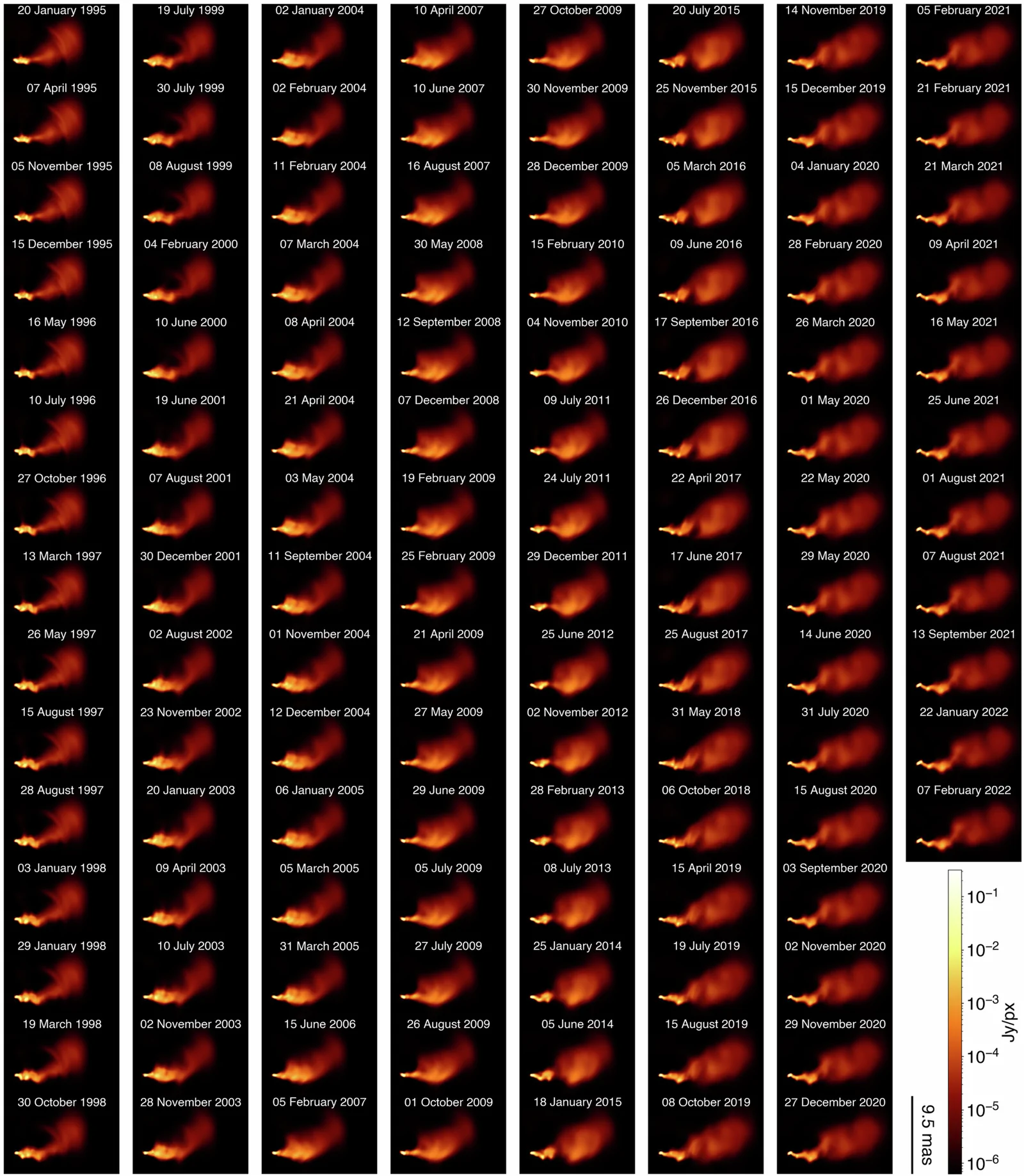
Video reconstruction of 3C 345, total intensity. All 116 total intensity frames of the dynamic imaging reconstruction of blazar 3C 345, from MOJAVE observations with the VLBA at 15 GHz, by the kine method. The x and y axis of the frames correspond to RA and Dec coordinates respectively, with the scale indicated at the bottom right of the figure.

**Extended Data Fig. 2:**
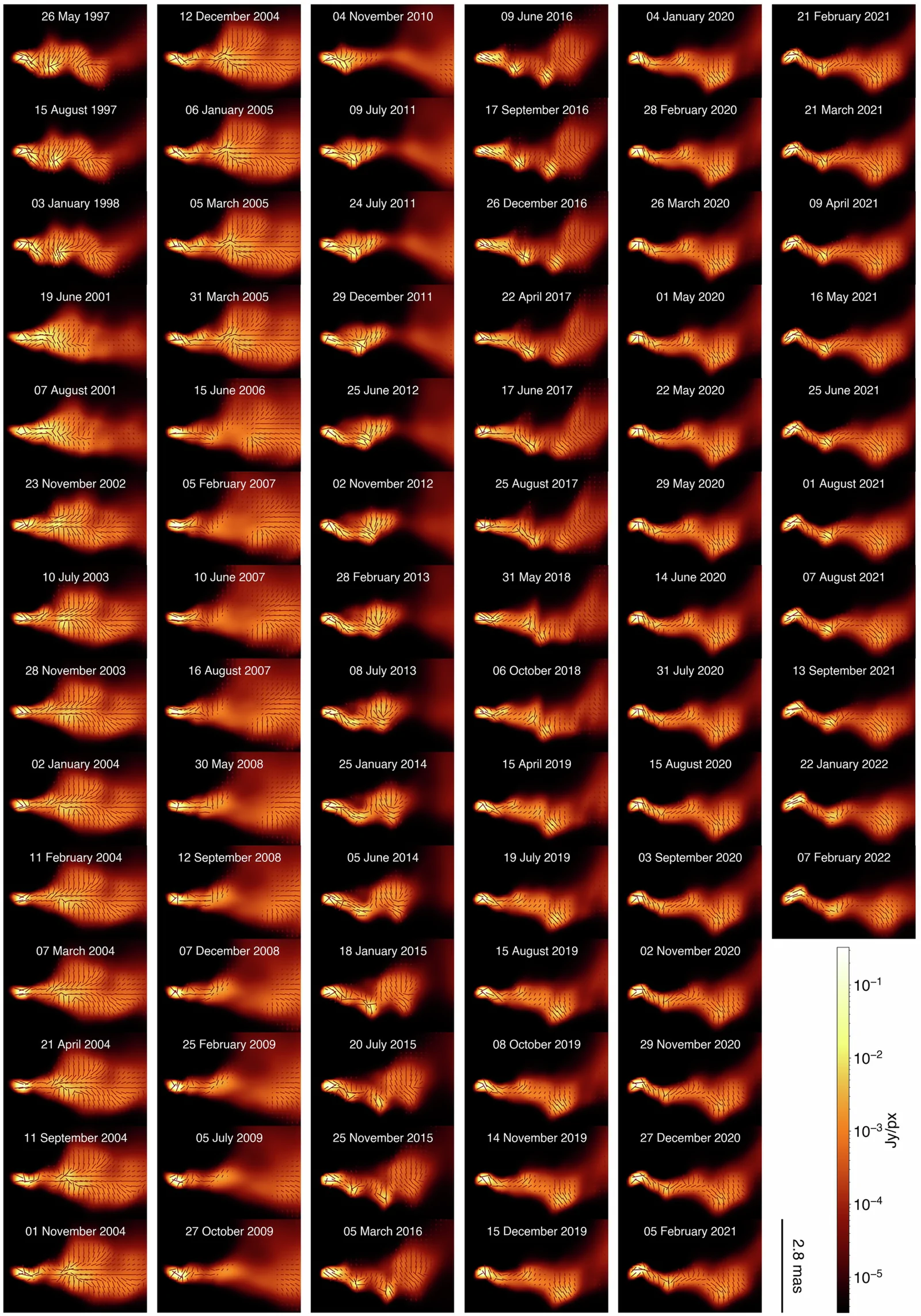
Video reconstruction of 3C 345, polarization. All 116 linear polarization intensity frames of the dynamic imaging reconstruction of blazar 3C 345, from MOJAVE observations with the VLBA at 15 GHz, by the kine method. The shown field of view is restricted to the inner jet to highlight the details of the linear polarization vector field. The x and y axis of the frames correspond to RA and Dec coordinates respectively, with the scale indicated at the bottom right of the figure.

The simultaneous imaging of all epochs with kine achieves a resolution of about 113 micro-arcseconds (μas), corresponding to a four-fold gain over the nominal value. The total achieved dynamic range is around 5 × 10^5^, about 140× more than traditional methods, which is due in part to the resolution improvement (approximately 14×) and in part to simultaneous imaging (about 10×). Figure 1 shows example frames out of the 116 video frames reconstructed (top rows). The full video is available in Extended Data Figs. 1 and 2 and Supplementary Information Video 1. The jet presents an inner jet, propagating westwards from a bright compact core, and a diffuse plume bending northwards, for a total extension of about 13 mas. Figure 1 (bottom rows) shows a magnification of the inner jet for three frames, with polarization ticks (left) and the plasma velocity vector field (right). From the jet core, plasma is continuously expelled downstream with occasional and noticeable ejection of discrete bright features, which initially move ballistically, before turning towards the jet axis, around 2–3 mas from the core, and vanishing into the plume (see, for example, the westernmost feature in Fig. 1b). These proper motions have been identified also in previous works through Gaussian model fitting at 15 GHz and other frequencies^21,29,30^. We focus our analysis on the jet plasma kinematics as an example of the possible studies enabled by our method. Supplementary Information includes further analysis of the jet direction variability.

### Jet velocity field

**Fig. 2:**
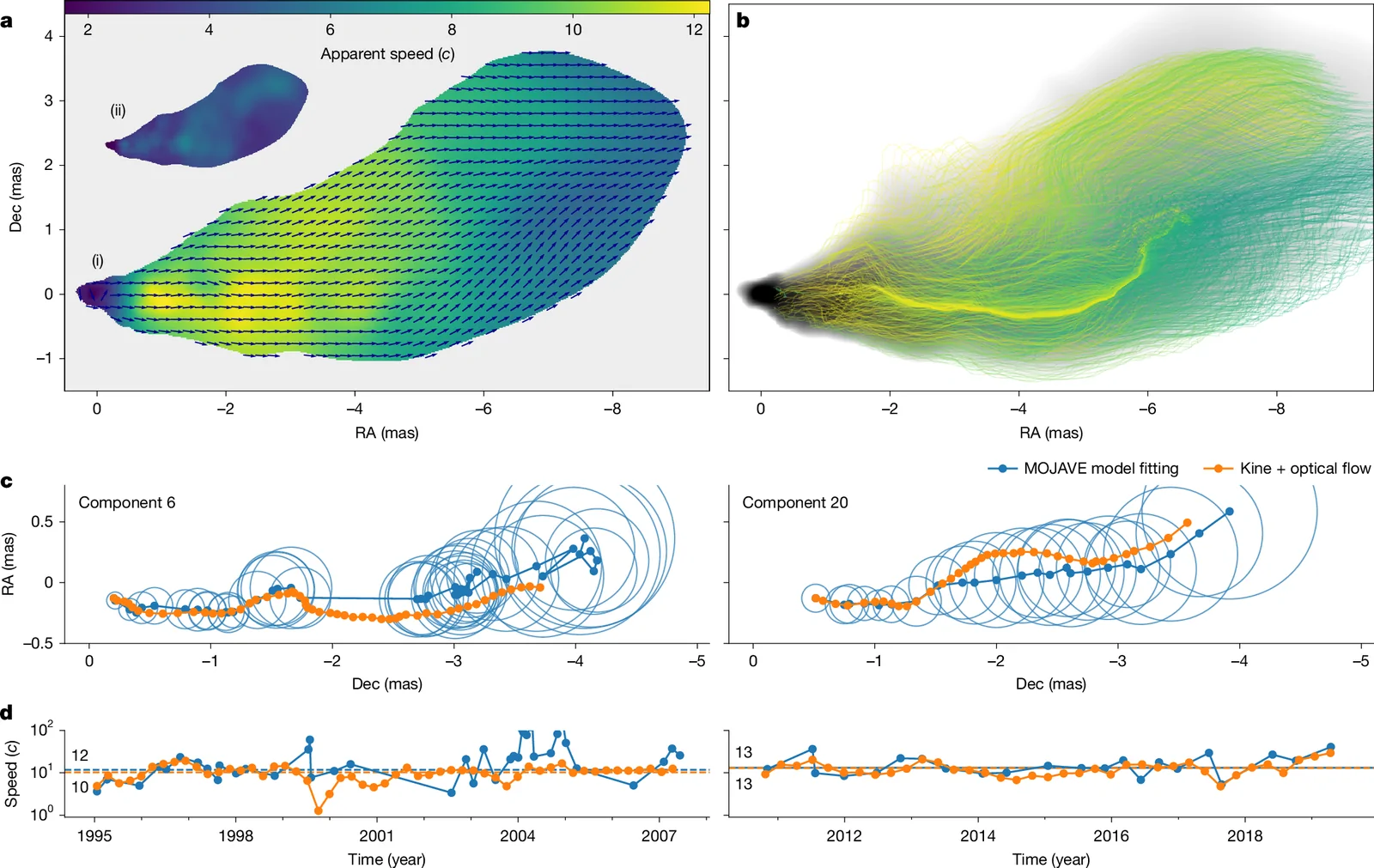
Optical flow velocity field in the jet plasma. **a**, Time average (i) and standard deviation (ii) of the apparent velocity, with magnitude indicated by the colour and direction by the arrows. **b**, Trajectories obtained by integrating the optical flow starting from May 2002 and representing the motion of test particles following the velocity vector field. The colour of each trajectory indicates the average speed of the trajectory, with the same scale as in **a**. **c**, Comparison of component trajectories from traditional model fitting (blue)^21^ and optical flow (orange). Blue circles indicate the size of the model-fitted components. **d**, Component speed compared with time, with average speed shown as a dotted line. The velocities reported in ref. ^21^ are *β*_app,C6_ = 11.79 ± 0.19*c* and *β*_app,C20_ = 13.16 ± 0.17*c*.

In contrast to the standard VLBI kinematic analysis, which relies on fitting Gaussian components to broad features travelling along the jet, we track the instantaneous, local velocity field of the plasma of the jet through optical flow estimation. This is enabled by the higher angular resolution of the reconstructions of our method, because of which we can obtain a velocity vector field, defined over space and time, at the same resolution as the video. Moreover, the time continuity of the video allows for motion-preserving interpolation between observed frames, circumventing the issue of irregular observation cadence. The instantaneous apparent velocity in the jet is shown in Fig. 1 (left), whereas in Fig. 2a, we present the time-average speed (i) and standard deviation (ii). By integrating the optical flow field from a given initial position, we obtain the trajectories followed by volume elements of the jet plasma, as shown in Fig. 2b. The optical flow trajectories following discrete components in the kine video agree with those obtained from model fitting of lower-resolution Gaussian components^21^ (Fig. 2c), which confirms the reliability of the optical flow method. However, the optical flow is not limited to tracking discrete bright components such as model fitting or wavelet decomposition. Owing to the high resolution of the video of kine and the locality of the optical flow vector field, it can also measure the velocity of any point in the jet, interpreted here as the velocity of the bulk jet flow in the absence of a passing component. Distinguishing between the speed of components and the speed of the flow is not possible with either Gaussian fitting or wavelet decomposition analysis. Details about frame interpolation with kine, a comparison between optical flow and model fitting and the optical flow limitations are discussed in the Supplementary Information.

From the optical flow measurements (Fig. 2d), the highest value of the mean apparent flow speed decreases from *β*_app_ = 12 ± 0.2*c*, between 1 mas and 3 mas from the core in the southern part of the jet, in which most of the components are ejected, to [9–11] ± 0.2*c* within the first 5 mas and [5–8] ± 0.2*c* further out in the diffuse emission. The maximum average speed corresponds to a physical speed of 0.997*c* for reported viewing angles. The speed standard deviation has no substantial location dependence and ranges between 3*c* and 6*c*, indicating that the plasma flow is turbulent and subject to relevant changes in apparent speed.

The velocities of the bright components (10–13*c*) are comparable with the average bulk plasma speed (9–12*c*) in the same region. This suggests that the components are not strongly shocked regions in the plasma flow, as previously proposed^16,31^, but more likely localized over-densities in the plasma or regions with increased emissivity. In the presence of strong shocks, we would expect a significant difference between the bulk velocity of the shocked material and the pattern velocity of the shock, which is not the case for this source. Nevertheless, it is worth noting that similar values for the shock pattern speed and the plasma flow speed can also arise if protons are not highly relativistic^32^. We further test our hypothesis by analysing the polarimetric properties of the bright features.

### Polarization and magnetic field

**Fig. 3:**
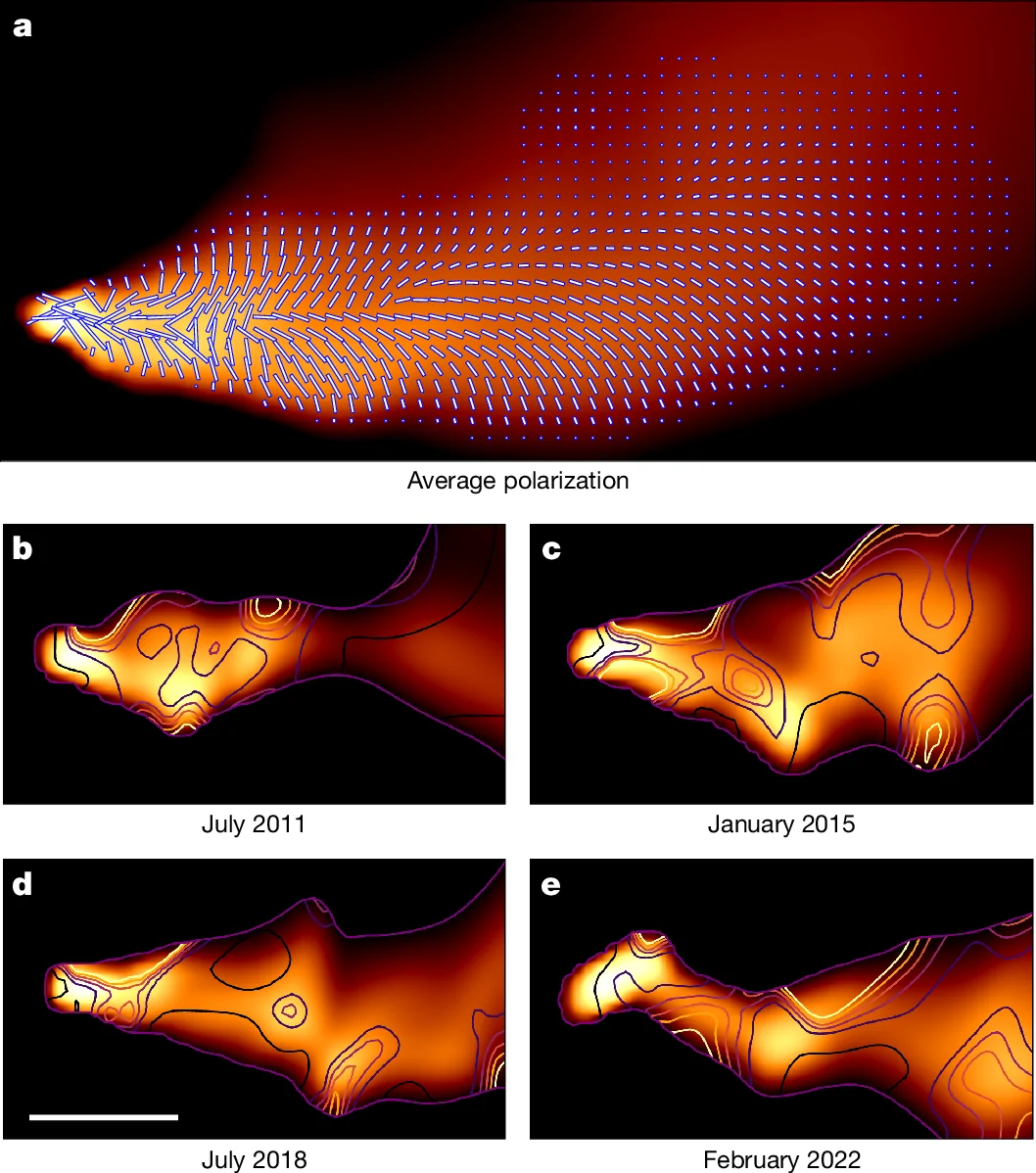
Polarization field structure in the 3C 345 jet. **a**, Time-average polarization, shown as ticks whose direction indicate the EVPA and length proportional to the logarithm of the linear polarization intensity. Owing to missing polarimetric data between 1995 and 2000, only frames from June 2001 onwards were included in the time average. Background image shows the time-average total intensity in colour scale. **b**–**e**, Instantaneous frames with total intensity shown as colour map and fractional polarization as contour lines. Contours are shown for *m* = 1%, 10%, 30% and 50 %, with darker contours corresponding to lower *m*. Scale bar, 1 mas (**b**–**e**).

kine reconstructions yield videos of the Stokes parameters ${\mathcal{I}}$, ${\mathcal{Q}}$ and ${\mathcal{U}}$, showing the evolution of the linear polarization $P={\mathcal{Q}}+i{\mathcal{U}}$ and fractional linear polarization ${m}_{{\ell }}:= | P| /{\mathcal{I}}$. Figure 1 shows the polarization field for three example frames, whereas Fig. 3a shows the time-averaged polarization field. An electric vector position angle (EVPA) structure in which the EVPAs align with the jet along the spine and orient perpendicularly near the jet border, recurs across multiple epochs (Fig. 1a) and is persistent also in the time-average polarization. When bright features are ejected, this pattern is disrupted as the feature travels through (Fig. 1b,c).

The EVPA structure is similar to the lower resolution polarization map reported in ref. ^33^ and is consistent with a long-lasting toroidal magnetic field threading the jet in 3C 345 (refs. ^34–36^), potentially resulting from a large-scale helical field dominated by its toroidal component^37^.

Fractional polarization maps offer another probe into the nature of the travelling bright features. Figure 3b–e shows four such features, overlaid with instantaneous *m*_*ℓ*_ contours. If the features were propagating shocks, we would expect locally higher *m*_*ℓ*_ at their location^16^. Instead, although the passage of a bright feature produces a slight local disruption of the underlying polarization pattern, the peaks in *m*_*ℓ*_ show no spatial correlation with the features in total intensity.

The absence of this correlation further disfavours strong shocks in the jet. A travelling shock compresses the plasma, amplifying the magnetic field component parallel to the shock front because of the increased density. In a turbulent or toroidal magnetic field, the compression enhances field ordering and increases *m*_*ℓ*_ within the shocked region, whereas a predominantly poloidal field would produce the opposite effect^16,38^. As our polarization pattern points to a toroidal magnetic field, if the bright components were shocks, we would expect localized increases in *m*_*ℓ*_, yet this is not observed. Combining this with the pattern and bulk speed comparison, we interpret the bright, compact features as regions of enhanced emissivity associated with locally amplified magnetic fields, in which plasma turbulence increases the magnetic pressure and Doppler boosting further enhances their brightness when ejected southwards.

## Discussion

The analysis conducted in this work was enabled by two properties of kine. First, simultaneous imaging of all observations allows information to be shared across frames, improving resolution and dynamic range beyond what is achievable by frame-by-frame imaging (Supplementary Fig. 8). Second, the neural representation produces a smooth, continuous model of the flux density, sampleable at any time coordinate, enabling continuous and local motion analysis by optical flow. Applied to multi-epoch observations, kine can provide marked advances in jet kinematics studies: from high-resolution time-continuous videos, it becomes possible to measure the instantaneous local velocity field rather than only tracking model-fitted components. Future application to other blazar sources may lead to a reevaluation of the nature of assumed travelling shocks or to precise measurements of their pattern and flow speeds.

Multi-epoch blazar imaging is not the only possible application of kine. The algorithm was originally developed for horizon-scale single observations of Sagittarius A* with the Event Horizon Telescope (EHT). In that case, the source variability timescale is shorter than the full observation duration and the instantaneous coverage is too sparse to constrain individual frames, making dynamic imaging the only approach to resolve the variability of the source.

In static mode, kine achieves super-resolution comparable to recent regularized maximum likelihood and Bayesian methods (for example, eht-imaging^7^, Comrade^8^, Resolve^6^), improving over CLEAN^5^. In dynamic mode, unlike algorithms such as StarWarps^39^, eht-imaging^40^, ngMEM^41^, kine enforces correlations through implicit regularization rather than explicit morphological priors, making reconstructions robust and the algorithm source-agnostic. It also requires no careful tuning or expensive exploration of the regularization hyperparameters. Compared with dynamic Bayesian methods such as Resolve^42^, kine offers considerably shorter runtimes and better scalability to large datasets.

Generally, kine can image any VLBI dataset, single or multi-epoch, static or dynamic, in full polarization. It requires minimal expertise from the user, while implicit regularization limits human bias in the previous assumptions. Its ability to scale to large datasets with a short runtime makes it the only algorithm that can now reconstruct high-dynamic-range videos of multi-epoch observations. Beyond its potential for imaging dynamic sources, such as blazars, black holes or X-ray binaries, kine holds promise for VLBI tasks that require automation. Applications outside radio astronomy are also conceivable: MRI reconstruction poses formally the same problem as VLBI imaging, and kine could prove useful for imaging moving organs. Finally, the kine framework is naturally extensible to multi-frequency imaging by treating frequency as an additional input coordinate, making spectral index mapping a target for future development.

## Conclusions

**Fig. 4:**
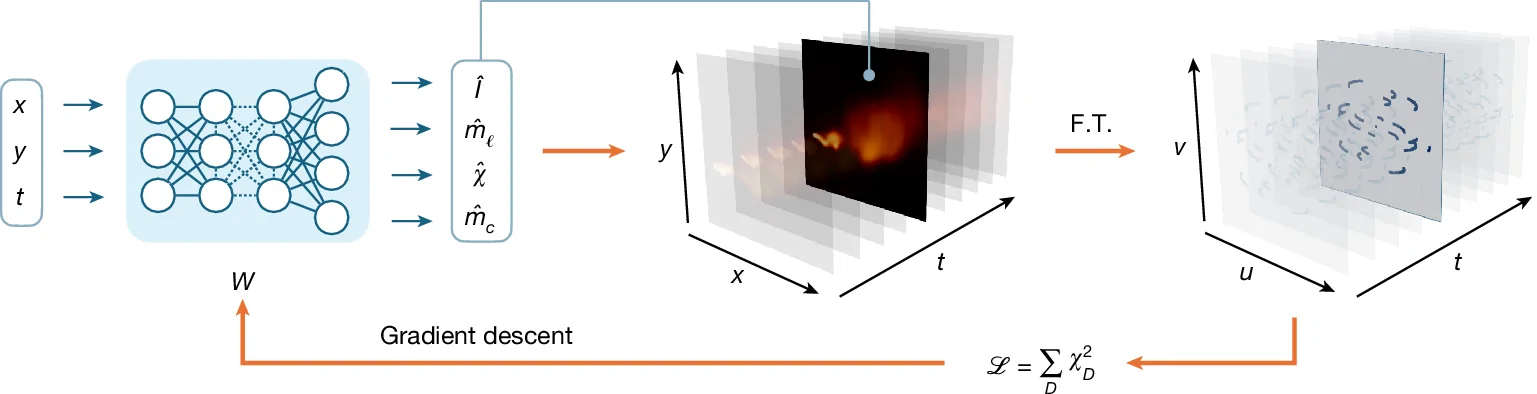
Diagram of one iteration of kine. The algorithm is composed of a forward model, a data constraint and an optimization method. The forward model consists of a coordinate-based multi-layer perceptron that maps space and time coordinates to the corresponding value of total intensity, linear polarization, polarization angle and circular polarization. A spatial Fourier transform is applied to the output video to compute the visibilities. The difference between the estimated and observed visibilities is computed through *χ*^2^s of the chosen data products, which are added in the loss function. The parameters of the multi-layer perceptron are optimized so to minimize the loss function. F.T., Fourier transform.

We presented a new dynamic imaging algorithm for VLBI observations of variable sources (Fig. 4) with an example application to multi-epoch observations of blazar 3C 345 from the MOJAVE program. The algorithm provides a time-continuous full-polarimetric video of the source at a resolution of about 113 μas and a dynamic range of approximately 5.1 × 10^5^. The super-resolution of kine is slightly higher than that of other algorithms, whereas the achieved dynamic range outperforms any available method because of the correlation of information across multiple observations. Combining our imaging results with optical flow results in a high-resolution map of the instantaneous projected velocity field in the source. For 3C 345, this allowed us to distinguish and compare the speed of discrete components and the average bulk flow speed.

We found no evidence that travelling bright components are strongly shocked regions, as previously proposed^16,31^, because the component speeds are of the same order as the average flow speed in the same regions. The shock interpretation is also disfavoured by the absence of correlation between the bright features and the peaks in the fractional polarization. Therefore, we interpret the travelling features as Doppler-boosted regions of enhanced emissivity associated with local increases in magnetic pressure. This could not be concluded from earlier studies^21,29^ because model fitting can only measure the speed of components, that is, the pattern speed of a possible shock, but not the underlying flow speed. Our results challenge only the current shock models for the 3C 345 blazar, but future systematic application of the proposed imaging and analysis technique to archival and future observations may produce more general results.

## Methods

The kine algorithm is a static or dynamic image reconstruction algorithm for interferometric data. It is a forward imaging method, based on a neural field representation^43^ of the brightness distribution of the source. In this section, we describe our methodology, starting with a general overview of VLBI measurements followed by an explanation of the kine imaging algorithm, which is shown in Fig. 4. Then, we describe the synthetic data used to test the performance of the algorithm and show the corresponding reconstructions. In the Supplementary Information, we present extensive tests validating the dynamic range, resolution, time interpolation and motion tracking of our method on synthetic datasets, of which we report the results here. We also present a performance comparison with the CLEAN method on real observations and the details of the optical flow velocity analysis.

### VLBI measurements

In radio interferometric observations, each antenna in the array records a signal proportional to the received electromagnetic flux density. By the van Cittert–Zernike theorem, the time-averaged correlation product of the signals recorded by any pair of antennas (called visibility) is the Fourier transform of the flux density spatial distribution on the sky plane, evaluated at a frequency proportional to the distance of the antennas. For a pair of antennas A and B, with projected baseline vector $\bar{b}=({b}_{x},{b}_{y})$, observing at wavelength *λ*, the ideal complex visibility *V*_AB_(*u*, *v*, *t*) is related to the flux density distribution ${\mathcal{I}}(x,y,t)$ by

1 $${V}_{\mathrm{AB}}^{({\mathcal{I}})}(u,v,t)=\int \int \,{{\rm{e}}}^{-2\mathrm{\pi i}(ux+vy)}\,{\mathcal{I}}(x,y,t){\rm{d}}x{\rm{d}}y,$$

where $(u,v)=\left(\frac{{b}_{x}}{\lambda },\frac{{b}_{y}}{\lambda }\right)$ are the *x* and *y* spatial frequencies (refer to ref. ^44^ for a detailed description of radio interferometry observations and imaging). The above equation, written for Stokes ${\mathcal{I}}$, holds for all Stokes visibilities $({V}_{\mathrm{AB}}^{({\mathcal{I}})},{V}_{\mathrm{AB}}^{({\mathcal{Q}})},{V}_{\mathrm{AB}}^{({\mathcal{U}})},{V}_{\mathrm{AB}}^{({\mathcal{V}})})$.

In practice, different sources of noise corrupt the measurement of visibilities. They are classified as baseline-dependent errors and site-dependent errors, so the measured visibilities can be expressed as

2 $${V}_{{\rm{AB}}}^{{\prime} }={G}_{{\rm{A}}}{G}_{{\rm{B}}}{{\rm{e}}}^{{\rm{i}}({\phi }_{{\rm{A}}}-{\phi }_{{\rm{B}}})}({V}_{\mathrm{AB}}+{{\epsilon }}_{\mathrm{AB}}),$$

where ${G}_{{\rm{A}},{\rm{B}}}{{\rm{e}}}^{{\rm{i}}{\phi }_{{\rm{A}},{\rm{B}}}}$ are the site-dependent errors in amplitude and phase, referred to as complex gains, and *ϵ*_AB_ is the thermal noise, which is Gaussian-distributed with baseline-dependent standard deviation^44^. The most problematic source of error is the complex gains, as they might be incorrectly estimated from the a priori calibration, whereas thermal noise can be fully characterized and incorporated in the loss function through the uncertainties *σ*_AB_ of the visibilities.

Therefore, although complex visibilities are the fundamental data product resulting from VLBI observations, the imaging process can use different data products that are constructed to be unaffected by site-dependent amplitude or phase corruptions. kine supports the following data products: complex visibilities *V*_AB_, amplitudes of the visibilities |*V*_AB_|, closure phases ${\varPhi }_{\mathrm{ABC}}:= \arg ({V}_{\mathrm{AB}}{V}_{\mathrm{BC}}{V}_{\mathrm{CA}})$, closure amplitudes *A*_ABCD_ ≔ |*V*_AB_*V*_CD_|/|*V*_AC_*V*_BD_| and the logarithm of closure amplitudes.

### Model

kine is a forward imaging algorithm for interferometric data, which models the polarized flux density distribution of a continuous image or video using a neural field^9^, parameterized by weights ${\mathcal{W}}$. The neural field is implemented as a coordinate-based neural network, specifically a multi-layer perceptron (MLP)^45^, which takes spatial coordinates (right ascension *x* and declination *y*) and time (*t*) as input, and outputs the total intensity flux density $\hat{I}$, fractional linear polarization ${\widehat{m}}_{{\ell }}$, electric vector position angle $\widehat{\chi }$ and fractional circular polarization ${\hat{m}}_{c}$ at each sky location and time, as shown in Fig. 4:

3 $${(\hat{{\mathcal{I}}},{\hat{m}}_{{\ell }},\hat{\chi },{\hat{m}}_{c})}_{{\mathcal{W}}}(x,y,t)={\mathrm{MLP}}_{{\mathcal{W}}}(x,y,t).$$

The hat symbol denotes estimated quantities, as opposed to the true or observed properties of the source. The Stokes parameters are then computed from the polarization quantities $(\hat{{\mathcal{I}}},{\hat{m}}_{{\ell }},\hat{\chi },{\hat{m}}_{c})$ using the transformation:

4 $$\left\{\begin{array}{l}\hat{{\mathcal{I}}}=\hat{{\mathcal{I}}},\\ \hat{{\mathcal{Q}}}=\hat{{\mathcal{I}}}\cdot {\hat{m}}_{{\ell }}\cdot \cos (2\hat{\chi }),\\ \hat{{\mathcal{U}}}=\hat{{\mathcal{I}}}\cdot {\hat{m}}_{{\ell }}\cdot \sin (2\hat{\chi }),\\ \hat{{\mathcal{V}}}=\hat{{\mathcal{I}}}\cdot {\hat{m}}_{c}.\end{array}\right.$$

We compute VLBI measurements from the predicted video using a fully differentiable forward model and optimize the MLP weights by minimizing a *χ*^2^ data-fit loss between the predicted and observed measurements. Within this forward model, estimated complex visibilities $\widehat{V}$ and associated data products are obtained by evaluating the 2D Fourier transform of the network-estimated flux density $\hat{{\mathcal{I}}}$ at the (*u*, *v*, *t*) points corresponding to the observation tracks. In practice, $\hat{{\mathcal{I}}}$ is evaluated on a discrete set of (*x*, *y*, *t*) points, and the visibilities are therefore computed using a discrete Fourier transform **F** implemented as a matrix multiplication. For each observation time *t*_*j*_ and *u*–*v* point *i*, the estimated visibilities are

5 $$\begin{array}{c}{\hat{V}}_{{\mathcal{W}}}({(u,v)}_{i},{t}_{j})=\sum _{k}{{\bf{F}}}_{ik}{\hat{{\mathcal{I}}}}_{{\mathcal{W}}}({(x,y)}_{k},{t}_{j}),\end{array}$$

where *k* runs over all spatial points at which the video is estimated, and both the estimated total intensity and the estimated visibilities are dependent on the network parameters ${\mathcal{W}}$.

MLP networks are known to exhibit a ‘spectral bias’^46^, by which training with gradient descent preferentially captures low spatial frequencies. In the context of VLBI imaging, this behaviour is advantageous, as it acts as an implicit regularizer that promotes smooth reconstructions and suppresses spurious high-frequency structure in both space and time. Moreover, the continuous nature of the neural representation enables the video reconstruction to be evaluated at times when no observations are available, by leveraging correlations learnt from neighbouring frames. This implicit regularization through neural representations has been successfully applied to other ill-posed inverse problems in astrophysical imaging, in which many possible solutions are consistent with a given set of measurements^47–49^.

### Optimization

We formulate the optimization as a noise-weighted data-fitting problem. Assuming Gaussian noise residuals in the data products, which is valid for visibilities and amplitudes and for closure quantities in the high-SNR regime, the natural loss function ${\mathcal{L}}$ is given by the sum of the *χ*^2^ contributions from the selected data products:

6 $${\mathcal{L}}=\sum _{D}{\chi }_{D}^{2}=\sum _{D}\left[\frac{1}{{N}_{t}}\mathop{\sum }\limits_{j=1}^{{N}_{t}}\frac{1}{{k}_{D}{N}_{D,j}}\mathop{\sum }\limits_{i}^{{N}_{D,j}}\frac{{({D}_{ij}-{\hat{D}}_{{\mathcal{W}}ij})}^{2}}{{\sigma }_{D,ij}^{2}}\right],$$

where *D* indicates a generic data product, the index *j* runs over all the observed times, from 1 to the total number of observed times *N*_*t*_; the index *i* runs over all data corresponding to the observed time *t*_*j*_, from 1 to the total number of data *N*_*D*,*j*_; and *k*_*D*_ is a normalization factor that takes into account the degrees of freedom of the data product. Any combination of one or more kinds of data products can be used in the loss function, and the loss function terms associated with each data product are weighted equally.

The optimization process consists of iterating the following steps until convergence. First, the algorithm estimates the video, with the current network parameters ${{\mathcal{W}}}_{k}$, on a coordinate grid with *x* and *y* regularly spaced and *t* spaced according to the observation times. Then it computes the loss function from the estimated video and the observed data. Finally, it updates the MLP parameters to ${{\mathcal{W}}}_{k+1}$, by applying a gradient descent optimization step of the loss function. More specifically, we use the Adamax optimizer^50^. In this work, convergence is considered to be reached when the moving average of the loss function over a window of 100 iterations does not decrease by more than 1% for at least 1,000 iterations, provided that all data products in the loss function have reached a value of about 1. This convergence was achieved after approximately 2 × 10^4^ iterations, a value that we finally set as the total number of iterations.

### Calibration-free imaging details

When imaging a dataset with no gain corruption, the best data products to use are complex visibilities, because they contain the full amount of available information. In that case, the imaging procedure in kine consists of running the optimization algorithm estimating all Stokes parameters simultaneously. However, if the dataset contains amplitude and/or phase gains, imaging must be performed using closure quantities. When imaging with closure or log-closure amplitudes, the information about the total flux of the frames is lost so a total flux regularizer term is added to the loss function, constraining the total flux in each frame to a value either provided by the user or computed from the instantaneous visibility of the shortest baseline. When imaging with closure phases, the information about the absolute location of the source in the frame is lost, so the reconstructed movie may show the source drifting smoothly across the frame. We solve this issue post-imaging by re-aligning the frames to a chosen feature, such as a jet core. The alignment procedure is detailed in the Supplementary Information.

The current version of kine does not model station gains simultaneously with the imaging. For the total intensity reconstruction, this problem is solved by relying on closure quantities. The closure quantities for the ${\mathcal{Q}}$ and ${\mathcal{U}}$ signals, however, are gain independent only in the absence of polarization leakage^51^, but, even in that case, the low SNR of the ${\mathcal{Q}}$ and ${\mathcal{U}}$ data, combined with the loss of information due to the use of closure quantities, may result in insufficient data constraints. Therefore, we found that the most effective procedure to produce a full-polarimetric video, in the presence of gain corruption, is to first image the data in total intensity only, using the appropriate closure quantities, then self-calibrate the data to the total intensity video reconstruction, and finally, image the self-calibrated data in full Stokes, using complex visibilities.

The kine imaging method was developed for video reconstruction of a variable source, but it can reduce to static imaging by removing the time dimension from the input coordinates.

### Architecture

kine is implemented in Python using the JAX deep learning framework^52^. We use a four-layer MLP for total intensity imaging and a deeper six-layer MLP for full-Stokes imaging of the self-calibrated data, both with 256 nodes per layer and a residual skip connection^53^ between the first and the last layers. The node outputs in all layers except the last are normalized with BatchNorm^54^ across the full set of input coordinates to have zero mean and unit standard deviation. Hidden layers use the Gaussian Error Linear Unit activation^55^, whereas the output layer uses SoftPlus for $\hat{{\mathcal{I}}}$ and a sigmoid for ${\widehat{m}}_{{\ell }}$, ${\widehat{m}}_{c}$ and $\widehat{\chi }$. Standard interferometry functions are handled by the eht-imaging library^7^. During optimization, spatial input coordinates are sampled on a regular grid, whereas the time coordinates follow the observation timestamps. For the imaging of 3C 345, we used 200 × 200 spatial input points with 75 μas spacing.

The runtime depends on the number of visibilities and epochs, the field-of-view to beam-size ratio, and the data products used. Optimizing the network on 116 epochs of 3C 345 data from the VLBA took approximately 1.3 h on four NVIDIA A100 GPUs, whereas imaging 4 days of M 87* EHT observations took around 20 min on a single A100.

### Initialization

When the (*u*, *v*)-coverage of the VLBI data is sufficiently dense, such as for VLBA observations, kine is able to reconstruct the image or video starting from a random initialization of the network weights. However, if imaging with closure phases, it may be useful to initialize the network to a simple shape, such as a Gaussian, to constrain the image to the correct location in the frame. We found that, although it is important that the initialization image has the correct total flux and covers approximately the area in which most of the flux of the source should be located, the specific shape of the initialization image does not affect the resulting image or video obtained when convergence is reached.

The initialization is carried out by optimizing the network directly on the initialization image ${{\mathcal{I}}}_{\mathrm{init}}(x,y)$, with a pixel-to-pixel distance loss function:

7 $${{\mathcal{L}}}_{\mathrm{init}}=\sum _{i,j}{({{\mathcal{I}}}_{\mathrm{init}}({x}_{i},{y}_{i})-{\hat{{\mathcal{I}}}}_{{\mathcal{W}}}({x}_{i},{y}_{i},{t}_{j}))}^{2}$$

without the computation of the image Fourier transform or other data products. After the weights of the network are optimized to output the initialization image, the optimization on the data products begins.

### Synthetic data validation

We validated the algorithm on multiple synthetic datasets prepared to resemble the morphology, coverage and noise of the real 3C 345 data. The first ground truth video consists of a geometric jet model in which the emission direction of the jet is precessing along a circular orbit, emitting ballistic components, which expand and decrease in brightness as they move away from the core. This results in a model with a continuous winding structure, which we refer to as model1. The second model, model2A, is constructed in a similar way, but with longer spacing between the emitted components, so that the model is composed of discrete, separate components emitted at different angles. The components have an initial full width at half maximum (FWHM) = 80 μas, which increases slightly as they move away from the core at a constant apparent speed of *β* = 11.4. The third model, model3A, is similar to the second but with the addition of a parabolic jet profile as a background to simulate a more realistic jet structure. We used these three models for the main validation tests, but we also prepared variations of model2A and model3A, with components of different sizes—FWHM = 60 μas for model2B and model3B and FWHM = 160 μas for model2C—and moving at different speeds—*β* = 22.7 for model2D and *β* = 5.7 for model2E—to test the resolution and motion recovery. For each observed epoch, we simulated a synthetic dataset from the model, using the corresponding real (*u*, *v*)-coverage and applying the same amount of thermal noise. We did not need to introduce complex gain corruption, because the closure quantities used in the first step of the imaging process are invariant under gain errors.

**Extended Data Fig. 3:**
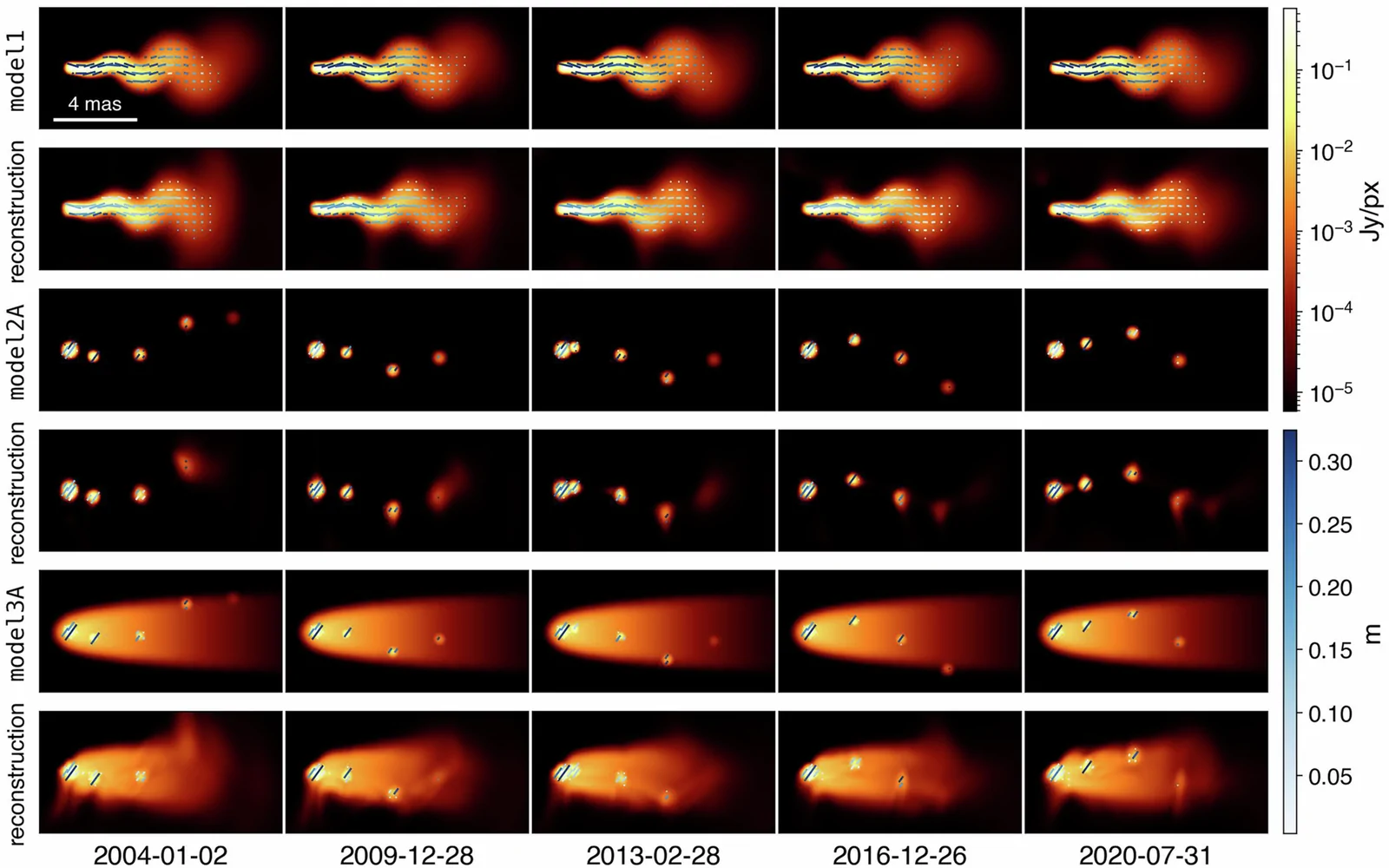
Ground truth models and video reconstructions with kine. Validation of the kine algorithm on synthetic data mimicking VLBA observations of 3C 345 at 15 GHz. The figure compares geometric jet model (rows 1,3,5) to the kine reconstructions (rows 2,4,6) of synthetic data generated from it. A few sample frames are shown as example. The ticks indicate the direction of the EVPA and their length is proportional to the logarithm of the linear polarization.

We imaged the synthetic data with the same parameters used for real data. Here, as an example, we present and discuss the reconstructions of model1, model2A and model3A. The ground truth videos are shown in the first, third and fifth rows of Extended Data Fig. 3, whereas the reconstructions of the synthetic data are in the second, fourth and sixth rows of the same figure. These show that kine is able to recover the models with high fidelity, which is reflected by the high values of the average normalized cross-correlation between the ground truth frames and the reconstructed ones (Extended Data Table 1).

In the Supplementary Information, we present the details of additional validation tests that quantify the dynamic range, super-resolution, component characterization, time interpolation and motion tracking achieved by our method, and we report the quantitative results of the tests in the subsequent sections. The improvements in reconstruction quality achieved by kine over traditional imaging methods, especially the dynamic range and resolution improvements, are dependent on the quality and quantity of the observations, so they should not be intended as intrinsic performance gains independent of dataset quality and temporal coverage.

Forward-modelling imaging algorithms can achieve higher resolution than traditional inverse methods by incorporating prior information. From the validation tests, we see that kine dynamic reconstructions have effective resolutions between about 100 μas and 125 μas, with an average of 113 μas, whereas kine static reconstructions have effective resolutions between about 110 μas and 145 μas, with an average of 125 μas. This shows that the main resolution improvement is achieved already at the static reconstruction level, with an improvement factor of 3.8 over the nominal resolution of 475 μas, whereas the dynamic reconstruction provides a marginally higher improvement factor of 4.2.

The dynamic range improvement of kine over traditional methods is a result of the combination of two factors: the resolution improvement of kine and the simultaneous imaging of multiple epochs. From the validation tests, we conclude that kine static reconstructions achieve a dynamic range between 6 × 10^3^ and 8 × 10^4^, whereas dynamic ones achieve a dynamic range from 2 × 10^5^ to 7 × 10^5^. This shows that dynamic imaging provides a higher dynamic range than static imaging. In this case, the improvement factor is of the order of about 10, which is compatible with the $\sqrt{N}=\sqrt{116}\simeq 11$ limit.

We tested the ability of kine to correctly resolve and locate small discrete components in the core region by fitting a double 2D Gaussian model on kine reconstructions. The tests show that components can be precisely located starting at about 110 μas from the core and tracked throughout their whole path. For a component fully detached from the core, the position angle can be recovered within 1° of the true value, whereas for those connected to the core, it is recovered correctly within 7°.

We tested the limit of the velocity that could be recovered with kine combined with the optical flow, and we conclude that we can accurately measure apparent velocities in the 3C 345 dataset up to at least approximately 23*c*, which exceeds the maximum speeds observed in the real data.

Owing to the continuous nature of neural field representations, kine reconstructions can be sampled at any arbitrary time, independent of the observed epochs. For data with the coverage and quality of 3C 345 observations, kine provides good, motion-preserving time interpolation for frames up to 6 months apart from the nearest observations. In the present work, we use time-interpolated frames in the optical flow analysis to have a video sampled at regular intervals. The longest separation between an interpolated frame and the nearest observation is 5.7 months, which is within the range of reliable interpolation.

### Comparison of CLEAN and kine

As shown from the validation tests, even when the instantaneous coverage is sufficient for static imaging, dynamic reconstructions achieve higher resolution and dynamic range than static ones. Here, we estimate the effective resolution and dynamic range of the static and dynamic reconstructions of kine of the real 3C 345 observations and compare them with images obtained with CLEAN. Supplementary Fig. 8 shows an example frame comparing traditional static imaging with CLEAN, snapshot static imaging with kine and dynamic imaging with kine. Both kine reconstructions show consistency with the CLEAN image, although they are able to resolve considerably more structure in the jet. As we do not have a ground truth in this case, we can estimate the dynamic range only with the traditional definition, while we estimate the resolution using the power spectrum comparison with the CLEAN model convolved with the nominal beam.

We obtain an average dynamic range of 3.6 × 10^3^ for CLEAN, 4.9 × 10^4^ for kine static and 5.1 × 10^5^ for kine dynamic, resulting in an improvement of one order of magnitude for kine static over the traditional CLEAN imaging method and two orders of magnitude for kine dynamic. The dynamic ranges of kine static have a wide distribution of values, whereas that of kine dynamic varies less. This means that kine dynamic is effective in improving the reconstruction quality of epochs with lower data quality by leveraging the information from previous and subsequent epochs.

We obtain a resolution of 141 μas for kine static and a resolution of 106 μas for kine dynamic, representing an improvement factor of 3.4 for kine static and 4.5 for kine dynamic with respect to CLEAN, whose average nominal resolution of 475 μas is set by the diffraction limit. These results are consistent with the estimates from the tests on synthetic data, although we consider the resolution estimate from real data slightly less reliable than that from synthetic data because the comparison is not carried out with the ground truth but rather with the CLEAN reconstruction. Therefore, we prefer to rely on the more conservative estimate from synthetic data for our conclusions.

## Online content

Any methods, additional references, Nature Portfolio reporting summaries, source data, extended data, supplementary information, acknowledgements, peer review information; details of author contributions and competing interests; and statements of data and code availability are available at https://doi.org/10.1038/s41586-026-10988-5.

## Supplementary information

Supplementary Information This file contains Supplementary Information sections 1.1–1.10, including Supplementary Figs. 1–10, Supplementary Tables 1–4 and Supplementary References. The sections include additional details on the analysis of the 3C 345 jet direction variations; optical flow velocity estimation technique and its comparison with model fitting for 3C 345; the post-imaging aligning procedure; validation tests aimed at establishing the performance of kine in terms of dynamic range, resolution, component tracking, velocity estimation and time interpolation, on synthetic data; a comparison of kine and CLEAN in terms of dynamic range and resolution in the specific case of 3C 345 observations; and an example of kine applied to EHT observations of M 87.

Supplementary Video 1 Evolution of 3C 345 over 27 years, from video reconstruction with kine. The main panel shows the total intensity video reconstruction of the 3C 345 jet obtained with kine from 116 VLBA observations. The second panel shows the polarimetric reconstruction in the inner portion of the jet. The third panel shows the projected velocity field in the inner portion of the jet.

## Figures and Tables

**Extended Data Table 1:**
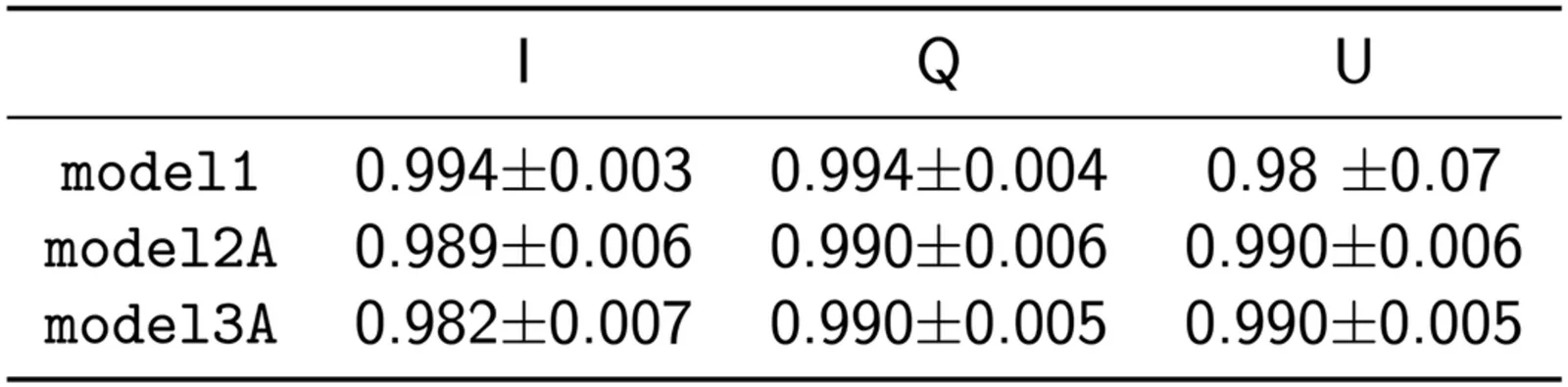
Model-reconstruction cross correlation

Average and standard deviation over frames of the cross correlation between the ground truth model and kine reconstruction.

## Data Availability

The observed data used in the imaging of 3C 345 are publicly available on the MOJAVE website (https://www.cv.nrao.edu/MOJAVE/index.html). The kine video reconstruction, the synthetic data, the post-imaging analysis and plotting notebooks are available at GitHub (https://github.com/mariannafoschi/kine3c345/).

## Code availability

The specific version of the kine algorithm used in this work is publicly available at GitHub (https://github.com/mariannafoschi/kine3c345/).

## References

[1] Blandford, R. D. & Znajek, R. L. Electromagnetic extraction of energy from Kerr black holes. *Mon. Not. R. Astron. Soc.* **179**, 433–456 10.1093/mnras/179.3.433 (1977).

[2] Blandford, R. D. & Payne, D. G. Hydromagnetic flows from accretion discs and the production of radio jets. *Mon. Not. R. Astron. Soc.* **199**, 883–903 10.1093/mnras/199.4.883 (1982).

[3] Narayan, R., Igumenshchev, I. V. & Abramowicz, M. A. Magnetically arrested disk: an energetically efficient accretion flow. *Publ. Astron. Soc. Jpn.* **55**, L69–L72 (2003).

[4] Tchekhovskoy, A., Narayan, R. & McKinney, J. C. Efficient generation of jets from magnetically arrested accretion on a rapidly spinning black hole. *Mon. Not. R. Astron. Soc. Lett.* **418**, L79–L83 (2011).

[5] Högbom, J. A. Aperture synthesis with a non-regular distribution of interferometer baselines. *Astron. Astrophys. Suppl. Ser.* **15**, 417–426 (1974).

[6] Junklewitz, H., Bell, M. R., Selig, M. & Enßlin, T. A. Resolve: a new algorithm for aperture synthesis imaging of extended emission in radio astronomy. *Astron. Astrophys.* **586**, A76 (2016).

[7] Chael, A. A. et al. Interferometric imaging directly with closure phases and closure amplitudes. *Astrophys. J.* **857**, 23 (2018).

[8] Tiede, P. Comrade: composable modeling of radio emission. *J. Open Source Softw.* **7**, 4457 (2022).

[9] Xie, Y. et al. Neural fields in visual computing and beyond. *Comput. Graph. Forum* **41**, 641–676 (2022).

[10] Lister, M. L. et al. MOJAVE. XV. VLBA 15 GHz total intensity and polarization maps of 437 parsec-scale agn jets from 1996 to 2017. *Astrophys. J. Suppl. Ser. ***234**, 12 (2018).

[11] Oei, M. S. S. L. et al. Black hole jets on the scale of the cosmic web. *Nature* **633**, 537–541 (2024). 10.1038/s41586-024-07879-y 39294348

[12] Cohen, M. H. et al. Radio sources with superluminal velocities. *Nature* **268**, 405–409 (1977).

[13] Vermeulen, R. C. & Cohen, M. H. Superluminal motion statistics and cosmology. *Astrophys. J.* **430**, 467–494 (1994).

[14] Gómez, J.-L., Marscher, A. P., Alberdi, A., Jorstad, S. G. & García-Miró, C. Flashing superluminal components in the jet of the radio galaxy 3C120. *Science* **289**, 2317–2320 (2000). 10.1126/science.289.5488.2317 11009410

[15] Jorstad, S. G. et al. Polarimetric observations of 15 active galactic nuclei at high frequencies: jet kinematics from bimonthly monitoring with the very long baseline array. *Astron. J.* **130**, 1418–1465 (2005).

[16] Marscher, A. P. & Gear, W. K. Models for high-frequency radio outbursts in extragalactic sources, with application to the early 1983 millimeter-to-infrared flare of 3C 273. *Astrophys. J.* **298**, 114–127 (1985).

[17] Raiteri, C. M. et al. Blazar spectral variability as explained by a twisted inhomogeneous jet. *Nature* **552**, 374–377 (2017). 10.1038/nature24623 29211720

[18] Fuentes, A. et al. Filamentary structures as the origin of blazar jet radio variability. *Nat. Astron.* **7**, 1359–1367 (2023).

[19] Horn, B. K. P. & Schunck, B. G. Determining optical flow. *Artif. Intel.* **17**, 185–203 (1981).

[20] Meinhardt-Llopis, E., Pérez, S. J. & Kondermann, D. Horn–Schunck optical flow with a multi-scale strategy. *Image Processing On Line* **3**, 151–172 (2013).

[21] Lister, M. L. et al. Monitoring of jets in active galactic nuclei with VLBA experiments. XVIII. Kinematics and inner jet evolution of bright radio-loud active galaxies. *Astrophys. J.* **923**, 30 (2021).

[22] Weaver, Z. R. et al. Kinematics of parsec-scale jets of gamma-ray blazars at 43 GHz during 10 yr of the VLBA-BU-BLAZAR program. *Astrophys. J. Suppl. Ser.* **846**, 98 (2022).

[23] Mertens, F. & Lobanov, A. Wavelet-based decomposition and analysis of structural patterns in astronomical images. *Astron. Astrophys.* **574**, A67 (2015).

[24] Steffen, W., Zensus, J. A., Krichbaum, T. P., Witzel, A. & Qian, S. J. A helical model for the compact jet in 3C345. *Astron. Astrophys.* **302**, 335–342 (1995).

[25] Unwin, S. C. et al. Superluminal motion in the quasar 3C 345. *Astrophys. J.* **271**, 536–550 (1983).

[26] Biretta, J. A., Moore, R. L. & Cohen, M. H. The evolution of the compact radio source in 3C 345. I. VLBI observations. *Astrophys. J.* **308**, 93–109 (1986).

[27] Lister, M. L. et al. MOJAVE. XVII. Jet kinematics and parent population properties of relativistically beamed radio-loud blazars. *Astrophys. J.* **874**, 43 (2019).

[28] Pötzl, F. M. et al. Probing the innermost regions of AGN jets and their magnetic fields with RadioAstron. IV. The quasar 3C 345 at 18 cm: magnetic field structure and brightness temperature. *Astron. Astrophys.* **648**, A82 (2021).

[29] Röder, J., Ros, E., Schinzel, F. K. & Lobanov, A. P. Up around the bend: a multiwavelength view of the quasar 3C 345. *Astron. Astrophys.* **684**, A211 (2024).

[30] Jorstad, S. G. et al. Kinematics of parsec-scale jets of gamma-ray blazars at 43 GHz within the VLBA-BU-BLAZAR program. *Astrophys. J.* **846**, 98 (2017).

[31] Blandford, R. D. & Königl, A. Relativistic jets as compact radio sources. *Astrophys. J.* **232**, 34–48 (1979).

[32] Marscher, A. P. Relativistic jets in active galactic nuclei. *AIP Conf. Proc.* **856**, 1–22 (2006).

[33] Pushkarev, A. B. et al. MOJAVE - XX. Persistent linear polarization structure in parsec-scale AGN jets. *Mon. Not. R. Astron. Soc.* **520**, 6053–6069 (2023).

[34] Lyutikov, M., Pariev, V. I. & Gabuzda, D. C. Polarization and structure of relativistic parsec-scale AGN jets. *Mon. Not. R. Astron. Soc.* **360**, 869–891 (2005).

[35] Fuentes, A., Gómez, J. L., Martí, J. M. & Perucho, M. Total and linearly polarized synchrotron emission from overpressured magnetized relativistic jets. *Astrophys. J.* **860**, 121 (2018).

[36] Fuentes, A., Torregrosa, I., Martí, J. M., Gómez, J. L. & Perucho, M. Magnetized relativistic jets and helical magnetic fields. II. Radiation. *Astron. Astrophys.* **650**, A61 (2021).

[37] Gabuzda, D. C., Reichstein, A. R. & O’Neill, E. L. Are spine-sheath polarization structures in the jets of active galactic nuclei associated with helical magnetic fields? *Mon. Not. R. Astron. Soc.* **444**, 172–184 (2014).

[38] Hughes, P. A., Aller, H. D. & Aller, M. F. Polarized radio outbursts in BL Lacertae. II. The flux and polarization of a piston-driven shock. *Astrophys. J.* **298**, 301–315 (1985).

[39] Bouman, K. L. et al. Reconstructing video of time-varying sources from radio interferometric measurements. *IEEE Trans. Comput. Imaging* **4**, 512–527 (2018).

[40] Johnson, M. D. et al. Dynamical imaging with interferometry. *Astrophys. J.* **850**, 172 (2017).

[41] Mus, A. & Martí-Vidal, I. New-generation maximum entropy method: a Lagrangian-based algorithm for dynamic reconstruction of interferometric data. *Mon. Not. R. Astron. Soc.* **528**, 5537–5557 (2024).

[42] Arras, P. et al. Variable structures in M87* from space, time and frequency resolved interferometry. *Nat. Astron.* **6**, 259–269 (2022).

[43] Mildenhall, B. et al. NeRF: representing scenes as neural radiance fields for view synthesis. In *Computer Vision – ECCV 2020* (eds Vedaldi, A., Bischof, H., Brox, T. & Frahm, J.-M.) 405–421 (2020).

[44] Thompson, A. R., Moran, J. M. & Swenson, G. W. Jr. *Interferometry and Synthesis in Radio Astronomy* 3rd edn (Springer, 2017).

[45] Popescu, M.-C., Balas, V. E., Perescu-Popescu, L. & Mastorakis, N. Multilayer perceptron and neural networks. *WSEAS Trans. Circuits Syst.* **8**, 579–588 (2009).

[46] Rahaman, N. et al. On the spectral bias of neural networks. In *Proc.**36th International Conference on Machine Learning* Vol. 97, 5301–5310 (PMLR, 2019).

[47] Levis, A., Chael, A. A., Bouman, K. L., Wielgus, M. & Srinivasan, P. P. Orbital polarimetric tomography of a flare near the sagittarius A* supermassive black hole. *Nat. Astron.* **8**, 765–773 (2024).

[48] Zhao, B., Levis, A., Connor, L., Srinivasan, P. P. & Bouman, K. L. Single view refractive index tomography with neural fields. In *Proc.**2024 IEEE/CVF Conference on Computer Vision and Pattern Recognition* 25358–25367 (IEEE, 2024).

[49] Zhong, E. D., Bepler, T., Berger, B. & Davis, J. H. CryoDRGN: reconstruction of heterogeneous cryo-em structures using neural networks. *Nat. Methods* **18**, 176–185 (2021). 10.1038/s41592-020-01049-4 PMC8183613 33542510

[50] Kingma, D. & Ba, J. Adam: A method for stochastic optimization. In *Proc. International Conference on Learning Representations* (ICLR, 2015).

[51] Roelofs, F. et al. Polarimetric geometric modeling for mm-vlbi observations of black holes. *Astrophys. J. Lett.* **957**, L21 (2023).

[52] Bradbury, J., Frostig, R., Hawkins, P., Johnson, M. J. & Katariya, Y. JAX: composable transformations of Python+NumPy programs (v.0.3.13). *GitHub.* http://github.com/jax-ml/jax (2018).

[53] He, K., Zhang, X., Ren, S. & Sun, J. Deep residual learning for image recognition. In *Proc. 2016 IEEE Conference on Computer Vision and Pattern Recognition* 770-778 (IEE, 2016).

[54] Ioffe, S. & Szegedy, C. Batch normalization: accelerating deep network training by reducing internal covariate shift. In *Proc. 32nd International Conference on International Conference on Machine Learning—Volume 37* (eds Bach, F. & Blei, D.) 448–456 (JMLR, 2015).

[55] Hendrycks, D. & Gimpel, K. Gaussian Error Linear Units (GELUs). Preprint at arxiv.org/abs/1606.08415 (2016).

