# Supplementary material for: Video reconstruction of variable VLBI observations with neural fields
Source: Nature. 2026 Aug 26;657(8133):900–5. doi: 10.1038/s41586-026-10988-5 (PMC13600935; doi:10.1038/s41586-026-10988-5)
Supplement: Supplementary file 1 [file 41586_2026_10988_MOESM1_ESM.pdf]

nature portfolio

https://doi.org/10.1038/s41586-026-10988-5

Supplementary information

Video reconstruction of variable VLBI observations with neural fields

In the format provided by the authors and unedited

Video reconstruction of variable VLBI observations with neural fields

Marianna Foschi[1,2,*,†], Brandon Zhao[3,†], Antonio Fuentes[1,†], Katherine L. Bouman[3], José L. Gómez[1], and Aviad Levis[4,5]

[1]Instituto de Astrofísica de Andalucía (CSIC), Spain
[2]Division of Physics, Mathematics and Astronomy, Caltech, USA
[3]Department of Computing and Mathematical Sciences, Caltech, USA
[4]Department of Computer Science, University of Toronto, Canada
[5]David A. Dunlap Department of Astronomy & Astrophysics, University of Toronto, Canada

[†]These authors contributed equally to this work.
[*]Corresponding author: foschimarianna@gmail.com

1 Supplementary Information

1.1 Irregular jet precession

The high-resolution of the kine video reconstruction of 3C 345 allows us to measure the direction at which components are ejected from the core. From each frame of video, we computed the jet launching direction angle by fitting an elliptical Gaussian to the core and identifying the angle with the orientation of the Gaussian's major axis. The direction is shown as a function of time in Supplementary Information Fig. 1, where blue points indicate the measured direction, while orange points show the average and standard deviation of a Gaussian Process regression to the data. The projected jet launching direction spans a projected angle of 60°, with intermediate smaller oscillations and with most material ejected towards -120° to -90° east of north. The variation of the direction angle is irregular and no significant periodicity is observed in the ejection direction.

Two different binary black hole (BBH) models [1] were proposed to explain the optical and radio variability of 3C 345 [2, 3]. The first one [2] assumes a precession period of 10 years and interprets the quasi-ballistic motion of superluminal features within 1 mas from the core as the result of a non-coplanar secondary black hole inducing torques in the inner parts of the disk. The second one [3], instead, consists of an equal-mass BBH system, resulting in a precession of the accretion disk axis with a 2570 years period. In this case, the yearly variability timescale observed in the light curve and component ejection process are interpreted as the result of thermal and mechanical instabilities and accretion rate variations.

What we observed instead is that the short-term variability of 3C 345 is directly linked to the change in the orientation of the jet core, which, nevertheless, we find to be irregular instead of periodic or quasi-periodic. The irregular jet precession was also observed in blazar 3C 84 [4] and could be attributed to instabilities in the accretion disk surrounding the central black hole or a misalignment between the angular momentum of the accretion disk and the black hole spin [5, 6, 7, 8, 9], which may cause a warping of the disk and a stochastic variation of the jet's direction. We note that an irregular, non-periodic variation of the apparent jet direction does not necessarily require changes in the launch orientation alone. Plasma instabilities developing within the relativistic jet itself, such as Kelvin–Helmholtz or current-driven (kink) modes, as well as asymmetric interactions with the surrounding medium, can also induce local distortions of the inner jet ridge line and brightness distribution that would manifest as stochastic changes in the measured core orientation. Our data

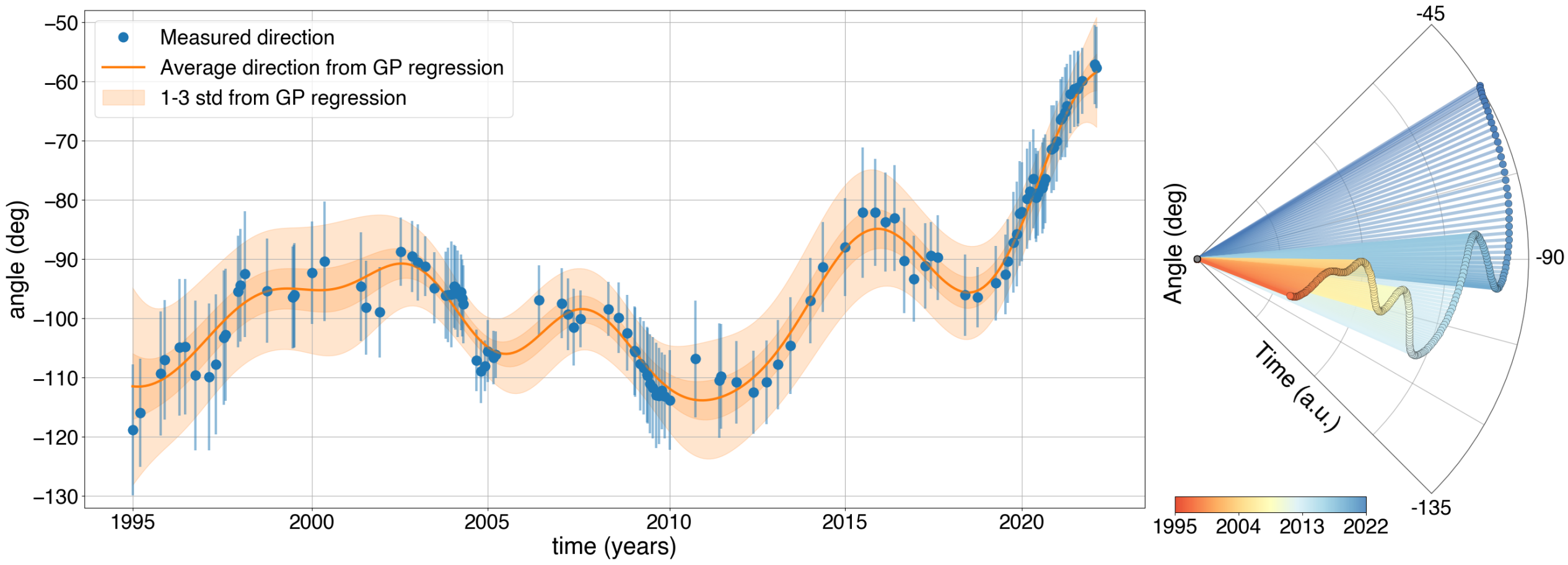

Figure 1: **Angle of the jet launching direction over time.** The convention used for the angle is 0 pointing north, with positive values in the north-west quadrant. (Left panel) Blue points indicate the angle computed from the frame corresponding to each observed epoch, with error bars indicating one sigma. The orange line indicates the mean of a Gaussian Process (GP) regression to the angle values, with the orange bands indicating one and three sigmas. (Right panel) The average of the GP regression is plotted in angular coordinates, for a more intuitive representation of the change in the launching direction. Red colors indicate the oldest epochs, while blue colors indicate the most recent epochs.

do not allow us to unambiguously distinguish between intrinsic variability at the jet base and such jet-side processes, but the absence of any clear periodicity disfavors models invoking a strictly periodic precession mechanism. Regardless of interpretation, the variation of the jet launching direction on a yearly timescale, shows that the short-term variability of the source is related to the irregular jet precession. Therefore the short-term irregular orientation changes should be incorporated in the variability models, which now only consider a long-term regular precession of the jet's orientation or assume a short-term periodic precession.

1.2 Optical Flow

Integrating the OF velocity field from the center of the first detection of a component yields trajectories that coincide with those obtained from fitting Gaussian components [10] as shown in the middle row of Fig. 2 in the main text. The trajectories of bright features tracked with OF and the trajectories of those same features obtained from model fitting are in great agreement within the inner ∼2 mas from the core, where the Gaussian components are well localized. Further downstream from the core, where model fitting struggles to constrain localized components in favor of diffuse ones, the two methods are in agreement on the overall trajectory, though small discrepancies are present. Likewise, the average component speeds from model fitting and the optical flow are also in agreement. We show a comparison for two example components, C6 and C20, in the lower panels of Fig. 2 in the main text, where the speed is plotted as a function of time. The optical flow analysis measures average speeds of $\beta_{\mathrm{app,C6}} = 10.3 \pm 0.2$ c and $\beta_{\mathrm{app,C20}} = 13.0 \pm 0.3$ c, which are similar to the previously reported values of $\beta_{\mathrm{app,C6}} = 11.79 \pm 0.19$ c and $\beta_{\mathrm{app,C20}} = 13.16 \pm 0.17$ c [10]. While these speeds are indeed similar, for component C6, model fitting and the optical flow yield a difference of approximately 1 c. We attribute this to the intrinsic differences in how these values are obtained (polynomial fitting of model fitted Gaussians in the visibility domain versus feature tracking in the image domain). For the other components, the OF velocity values have similar values that match the velocities obtained with model fitting. Given the agreement between the optical-flow motion field and the lower-resolution trajectories obtained from prior model fitting [10], the optical flow provides a reliable estimate of the apparent plasma flow speed.

OF aims to accurately capture the apparent motion of features in any region of the jet, matching the motion detected by human perception. However, because the algorithm relies on image gradients,

it can only track the motion of visible features. As a result, in the limit of a perfectly laminar flow the optical flow would report a null velocity, whereas in the presence of turbulence the inferred velocity is expected to be accurate. This explains why the velocity appears to be extremely low in the core and its proximity (right panels of Fig. 1 in the main text and top left panel in Fig. 2 in the main text). In the present case, we consider the OF to provide a reliable estimate of the jet velocity starting from ∼0.5 mas from the core. The velocity field map is also limited in resolution by the resolution of the reconstructed video, which in this case is $\sim 113\,\mu$as. Correlations that extend over the scale of the effective resolution should be interpreted as the result of actual coherent motion in the jet. The non-null velocity detected in the core region is caused by residual frame-to-frame misalignment. We consider the time average of the average velocity reported for pixels contained in the core, as the uncertainty on the individual instantaneous OF velocity measurements, since we believe this to be the limiting source of uncertainty. The uncertainties we report are of the same order of those obtained by component model fitting [10], showing that resolution is not the factor restricting the precision of the measurements, which is instead limited by the irregular evolution and motion of the jet.

1.3 Post imaging alignment

When imaging with closure phases, the information about the absolute location of the source in the frame is lost. Because of this, the reconstructed movie may show the source drifting smoothly across the field of view. We solve this issue post-imaging, by re-aligning the frames to a chosen feature, such as a jet core. The alignment process consists of shifting each frame by the amount that maximizes its cross-correlation in the core region with respect to the reference frame. More specifically we align frames according to the following steps:

1. We locate the peak brightness pixel in the core.
2. We mask out every pixel not included within a radius of 225 μas from the brightest pixel.
3. We blur the core with a 400 μas Gaussian beam so that no internal structure is distinguishable in the core.
4. We upscale the number of pixels in the image by a factor of 10 to enable finer discrete shifts.
5. We shift the frame by the number of pixels that maximizes the correlation between the current masked, blurred, and upsampled frame and the equally processed previous one.
6. We iterate the steps above for all frames.

1.4 Dynamic range tests

In VLBI, dynamic range is a measure of how faint of a structure the imaging method can reliably reconstruct, in comparison to the brightest ones. The dynamic range (DR) is conventionally defined as the ratio between the maximum value in the image ($I_{\max}$) and the image noise level, which here we defined as the root mean square error ($RMSE$) of the image in a portion not containing any real emission:

$$DR = \frac{I_{\max}}{RMSE(I_{\text{noise}})}\ . \tag{1}$$

`kine`'s dynamic range improvement is a result of the combination of two factors: `kine`'s resolution improvement and the simultaneous imaging of multiple epochs. If the resolution of an image increases, the maximum brightness of unresolved sources changes. Let's consider an unresolved point source, which is represented in the image by a Gaussian function $g_1(x, y) = A_1 \exp\left(-(x^2 + y^2)/2\sigma_1^2\right)$. By increasing the resolution of the image, e.g. by deconvolving with a Gaussian kernel, the point source will be represented with a narrower Gaussian $g_2(x, y) = A_2 \exp\left(-(x^2 + y^2)/2\sigma_2^2\right)$, while the total flux of the source remains constant. By equating the source's fluxes at the two different resolutions:

$\int g_1(x, y)\, dxdy = \int g_2(x, y)\, dxdy$, we obtain the relation between the peak brightness in the low resolution image and that in the high resolution image:

$$\frac{A_2}{A_1} = \left(\frac{\sigma_1}{\sigma_2}\right)^2 \tag{2}$$

Assuming that the background noise is dominated by thermal noise, and not imaging artifacts, the noise level remains unchanged by a resolution improvement. This happens because regions of extended emission or regions of no emission (i.e. regions where the emission is at the same level of noise) are already resolved and therefore will only see minor increases of the present modulations. For single-epoch static imaging, the fact that the noise level is unaffected by a resolution improvement is expected, since there is no additional information that could lower the noise level. As a result, the dynamic range is expected to improve by a factor equal to the square of the resolution improvement.

The DR also improves because of the simultaneous imaging of multiple epochs, under the reasonable assumption of independent noise realizations and comparable sensitivity across epochs. Noisy imaging artifacts don't correspond to any real structure in the source image and, since the (u, v)-coverage and noise change from epoch to epoch, they are not consistent from frame to frame and so they are not recovered in a time-regularized video, which requires frame-to-frame consistency. Additionally, under the assumption of frame similarity across the video, imaging multiple epochs together is similar to time averaging the data to improve the signal-to-noise ratio. Therefore, from multi-epoch imaging alone, we should expect a dynamic range improvement up to $\sqrt{N}$ where N is the number of epochs. In the case of a static unchanging source, we should expect a DR improvement equal to this limit, though for an evolving source we should expect a lower value.

We quantified the dynamic range achieved by `kine` on `model1` and `model3A`, which are the models with diffuse structure of varying flux density, for both dynamic imaging reconstructions and individual frame-by-frame "static" reconstructions. Estimating the dynamic range of our reconstructions using Eq. 1 is however not ideal. Indeed the definition is highly sensitive to the peak brightness in the image, which may be an unstable quantity and it is subject to the uncertainty in the selection of the area of no emission. Additionally, it is not straightforward to establish that the faint emission reconstructed in the supposedly dark parts of the image corresponds to the effective noise level. Our model does not impose sparsity priors nor constrain the emission in the central parts of the frame, so the modeled emission in the dark regions is unconstrained up to the effective noise threshold. Therefore, it is reasonable to believe that such emission is representative of the effective noise in the image. To ensure that that is the case, we compared the RMSE in the dark regions of the CLEAN images (which include residuals) and the RMSE from the same regions in the static `kine` images. The estimates yielded similar values (a median of 2×10^{-6} Jy/px for CLEAN and 3×10^{-6} Jy/px for static `kine`), which is what we would expect since, in the case of static imaging, there is no extra information that might reduce the effective noise level, while at the same time `kine`'s resolution improvement may marginally increase minor peaks in the dark areas. However, computing the noise floor from modeled quantities subject to spatio-temporal correlations is not ideal, which is why we paired the first estimate of the dynamic range with a second method that does not require an explicit definition of the noise level, but rather allows us to determine it in practice. Dynamic range is a measure of how faint the faintest structures that can be reliably detected are, compared to the brightest ones. Our second method provides that information by comparing the reconstructions with the ground truth. Specifically we rescale both the ground truth and the reconstruction images in the $[0, 1]$ interval in logarithmic scale, with a chosen dynamic range:

$$I \longrightarrow \frac{\log(I) - \log(I_{\min})}{\log(I_{\max}) - \log(I_{\min})} \quad , \quad \text{with} \quad \begin{cases} I_{\max} &= \max(I) \\ I_{\min} &= \max(I)/DR \end{cases} \tag{3}$$

and we clip image values outside the $[0, 1]$ interval. We then compute the cross correlation between the rescaled ground truth and reconstruction for different values of dynamic range. We expect the correlation to be high for lower DR values and to decrease for DR values above the reconstruction

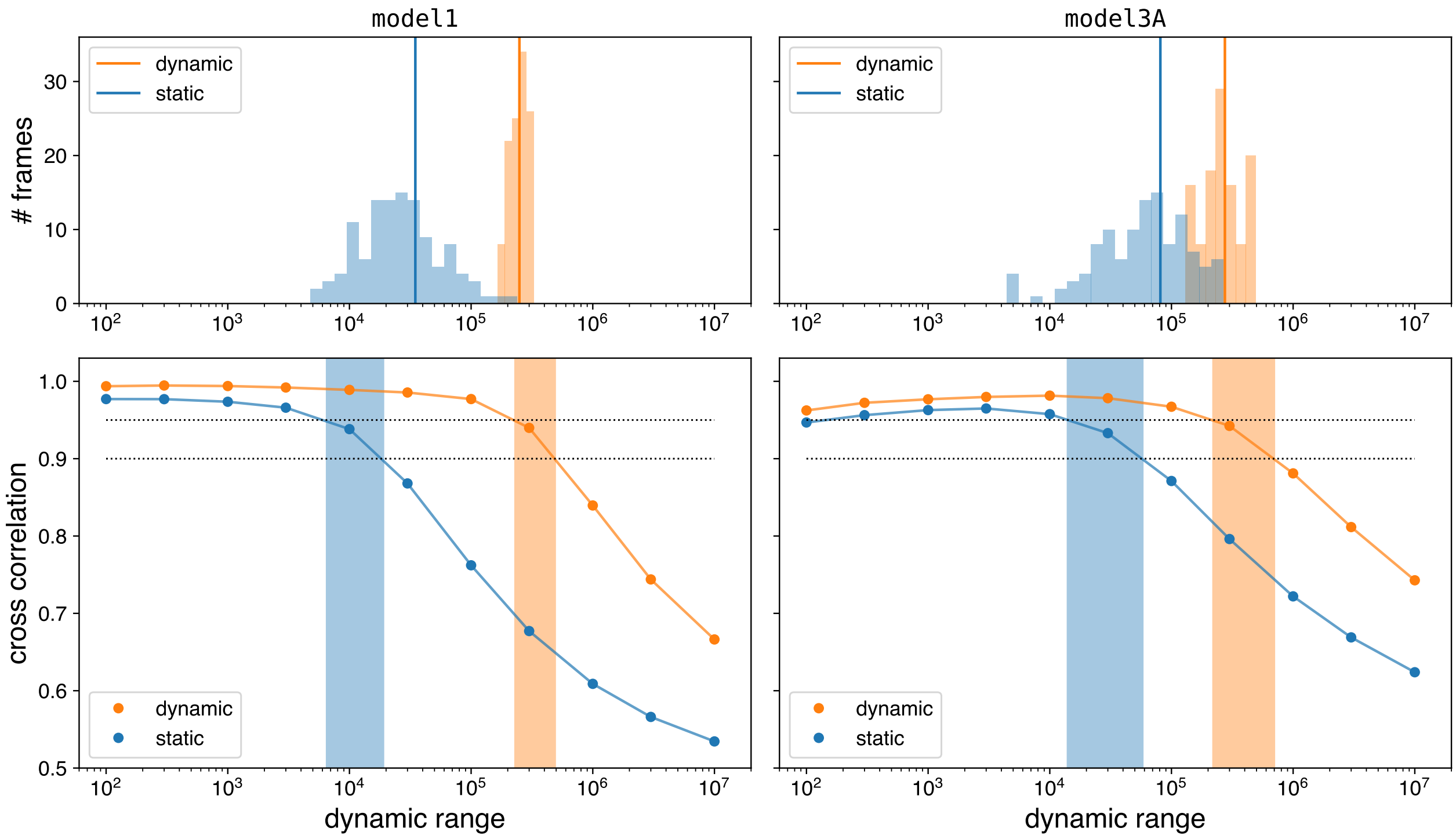

Figure 2: **Dynamic range tests.** Dynamic range of `kine` reconstruction of synthetic data from jet geometric models. (Top) Distribution across frames of the dynamic range computed with method 1 for `model1` (left) and `model3A` (right). The vertical lines indicate the average value. (Bottom) Cross correlation between reconstructed video and ground truth as a function of dynamic range for `model1` (left) and `model3A` (right). Vertical bands indicate the dynamic range computed with method 2, horizontal dotted lines mark the 0.9 and 0.95 cross correlation thresholds.

	dynamic		static	
	method 1	method 2	method 1	method 2
`model1`	2.5×10^5	$[2.3 \times 10^5, 4.9 \times 10^5]$	3.5×10^4	$[6.5 \times 10^3, 1.9 \times 10^4]$
`model3A`	2.7×10^5	$[2.2 \times 10^5, 7.0 \times 10^5]$	8.1×10^4	$[1.4 \times 10^4, 5.8 \times 10^4]$

Table 1: **Dynamic range tests.** Effective dynamic range of the `kine` reconstructions of synthetic data for static and dynamic reconstructions.

dynamic range, for which noise artifacts begin appearing in the rescaled image. So we estimate the dynamic range of the reconstruction as the value for which the cross correlation decreases below a set threshold. The advantages of this method are that it is not sensitive to fluctuations in the peak brightness of the image and does not depend on computing the noise level directly from the image. The downsides are the degree of arbitrariness in the choice of the threshold and the fact it requires a ground truth image for comparison.

In Supplementary Information Fig. 2 we present results from both methods. The first row of the figure shows the DR distribution across frames computed with method 1, for both the static and dynamic reconstruction, with the average DR marked with a vertical line. We observe that dynamic reconstructions not only provide an improvement in the average dynamic range but also a lower variance of the dynamic range from frame to frame, overcoming the occasional decrease in data quality, by propagating information from nearby frames. The second row displays the average cross correlation between reconstruction and ground truth as a function of the dynamic range. The decrease in cross correlation is gradual, so we considered values between 0.9 and 0.95 as suitable thresholds to determine the dynamic range hence providing a range of possible values for the DR, instead of a single value. The dynamic ranges of the `kine` reconstructions are reported in Table 1, from which we

can conclude that static reconstructions achieve a dynamic range between 6×10^3 and 8×10^4, while dynamic ones achieve a dynamic range spanning from 2×10^5 to 7×10^5. This shows that indeed dynamic imaging provides a higher dynamic range than static imaging. In this case the improvement is of a factor of $\sim$10, which is compatible with the $\sqrt{N} = \sqrt{116} \simeq 11$ limit.

1.5 Resolution tests

We chose two different methods to quantify the effective resolution achieved by `kine`'s reconstructions of synthetic data. Both methods rely on finding the optimal Gaussian kernel needed to blur the ground truth to match the resolution of the reconstruction. In the first method, we computed the 2D spatial power spectrum (PS) of each frame of the reconstruction. The power spectrum measures the strength of the image signal at different spatial scales and drops in correspondence of the smallest spatial scales resolved. To determine the resolution of the reconstruction, we blurred the model frames with Gaussian kernels of increasing FWHM and we computed the χ^2 between the PS of the blurred images and the PS of the corresponding reconstruction. The FWHM which resulted in the best χ^2 fit of the PS, was then added in quadrature to the reference resolution of the model images, i.e. the size of the smallest component in the true model, to obtain the effective resolution of the `kine` reconstructions. Finally we averaged the effective resolution across all epochs. In the second method, we blurred the model frames with Gaussian kernels of increasing FWHM and we computed the cross correlation between the blurred ground truth and the reconstruction. The FWHM for which the cross correlation peaks was added in quadrature to the reference resolution of the model images to obtain the effective resolution of the reconstruction. Also in this case we considered the average resolution across frames. We performed these tests on all the models with features of size equal or smaller than the expected resolution of the method, i.e. `model1`, `model2A`, `model3A`, `model2B`, and `model3B`, to test the limits of the achievable resolution. We performed these tests on both the dynamic and static reconstructions, to compare the resolution gain of dynamic imaging. The effective resolutions are reported in Supplementary Information Table 2, which shows that dynamic reconstructions have effective resolutions between $\sim$100 μas and $\sim$125 μas, with an average value of 113 μas, while static reconstructions have effective resolutions between $\sim$110 μas and $\sim$145 μas, with an average value of 125 μas. This shows that the major resolution improvement is achieved already at the static reconstruction level, with an improvement factor of 3.8 over the nominal resolution of 475 μas, while the dynamic reconstruction provides a marginally higher improvement factor of 4.2.

	dynamic			static			
	method 1	method 2	average	method 1	method 2	average	nominal
`model1`	128 μas	123 μas	125 μas	146 μas	148 μas	147 μas	-
`model2A`	106 μas	105 μas	106 μas	121 μas	114 μas	117 μas	-
`model3A`	113 μas	120 μas	117 μas	115 μas	121 μas	118 μas	-
`model2B`	102 μas	99 μas	101 μas	112 μas	105 μas	109 μas	-
`model3B`	107 μas	121 μas	114 μas	109 μas	150 μas	130 μas	-
average resolution	-	-	(113±8) μas	-	-	(124±13) μas	475 μas

Table 2: **Resolution tests.** Effective resolution of the `kine` reconstructions of synthetic data from different models.

1.6 Gaussian fitting tests

Since most of our analysis of the 3C 345 results relies on resolving the core region at scales smaller than the nominal beam and tracking components in the vicinity of the core, we conducted additional tests on `kine`'s ability to correctly resolve and locate small discrete components in the core region. We performed a Gaussian fitting on the reconstructions of `model2C`, `model2A`, and `model2B`, which are composed respectively of 60 μas, 80 μas, and 160 μas components ejected from a brighter 160 μas core and compared results with the same fitting procedure applied on the ground truth. Restricting

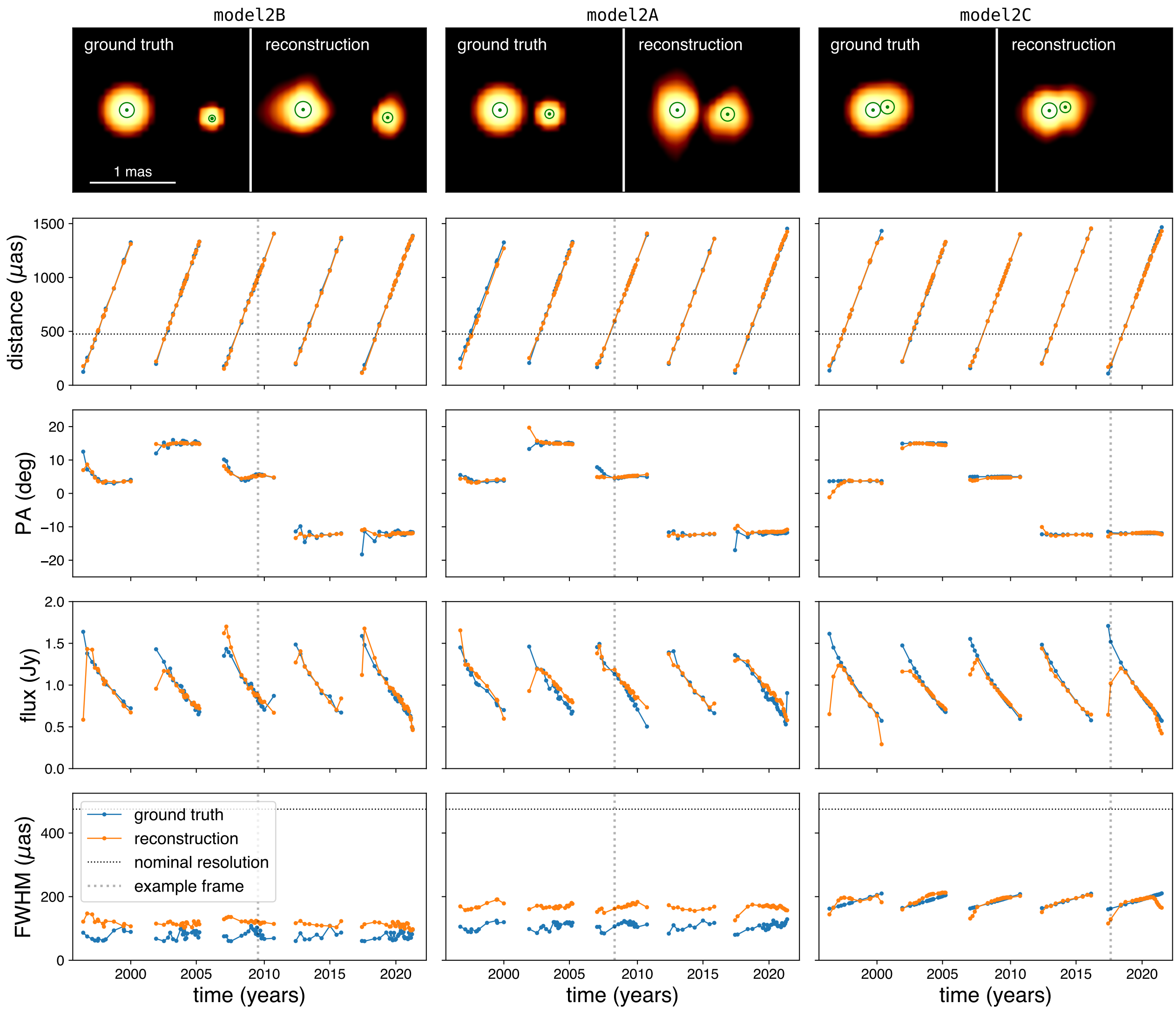

Figure 3: **Core region resolution and tracking test.** (Row 1) Example frames of the core region in the ground truth videos and the corresponding reconstructions, for three models with emitted components of different sizes. (Rows 2-5) Parameters of the Gaussian fit (distance from core, position angle PA, flux, and FWHM) for the moving component as a function of time, for both the ground truth video and the reconstruction. The vertical lines indicate the time of the example frame shown above. The horizontal lines mark the nominal resolution.

our focus to the core region, where at most two Gaussians (the core plus one component) are present at any given frame, we fitted a double 2D Gaussian model, using the FWHMs, total fluxes, and center coordinates as variable parameters. For the best fit results we computed the distance and position angle of the component with respect to the core.

The first row of Supplementary Information Fig. 3 shows an example frame of the ground truth and the reconstruction for each model, with the center and FWHM of the best fit Gaussians overplotted in green. The following rows present, for each model, the best fit parameters as a function of time, comparing the Gaussian fit performed on the reconstruction to that performed on the ground truth. From `kine` reconstructions, components can be precisely located starting at $\sim$110 μas from the core and tracked throughout their whole path for all models, including those whose components are smaller than the effective resolution achieved by `kine` (i.e. `model2C` and `model2A`). When the component is fully detached from the core, the position angle can be recovered within 1° of the true value, while before that, for smaller components in both the ground truth and the reconstruction, it is recovered correctly within 7°. In both cases, the error is well below the uncertainty on individual points in the measurement of the core's orientation (Fig. 1), showing that resolution is not a limiting factor

in the analysis. Before the components separate from the core, the flux is not always estimated correctly because the Gaussian fitting sometimes misattributes part of the flux between the core and the component, but for detached components, the flux is well recovered, with estimates within 0.1 Jy of the true value. For the components in `model2B`, which are larger than `kine`'s effective resolution, the FWHM is recovered faithfully, within ∼20 μas of the ground truth, while for `model2C` and `model2A`, the components' FWHM are overestimated. For `model2C` and `model2A` the components are so small that the measurement of the FWHM is noisy for both the ground truth and the reconstruction, but the reconstruction presents a clear bias in recovering larger components. Overall this test provides further confirmation that dynamic `kine`'s effective resolution is larger than 80 μas and smaller than 160 μas and shows that `kine` can accurately resolve and track features in the core region within the nominal beam, which in Supplementary Information Fig. 3 is indicated with a dotted line.

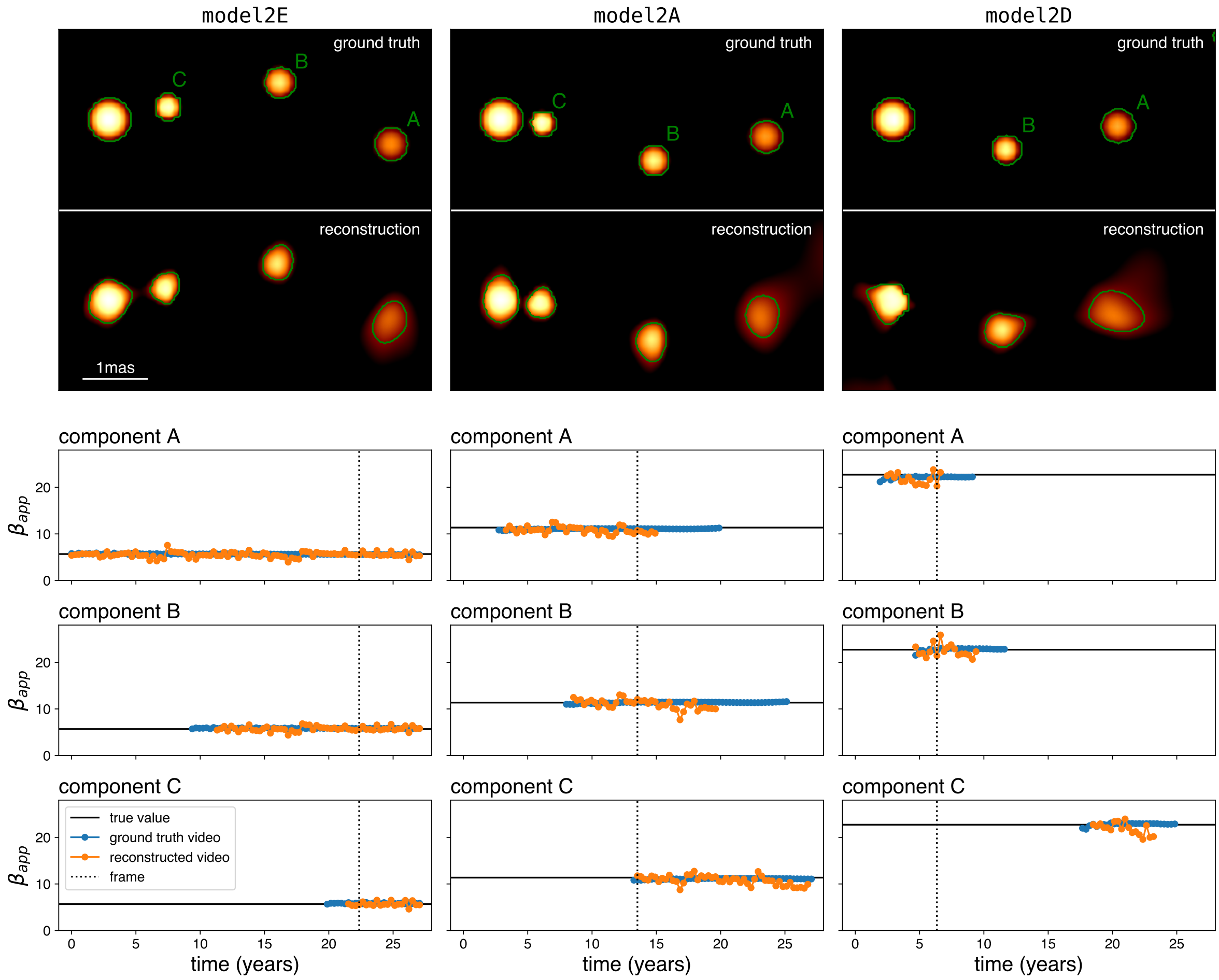

Figure 4: **Motion tracking validation test.** (Rows 1-2) Example frames of the ground truth videos and the corresponding reconstructions, for three models with different velocities. Green contours identify the discrete components. (Rows 3-5) Component apparent velocities as a function of time, measured with the optical flow method on the time interpolated ground truth and reconstructed videos. The vertical lines indicate the time of the example frames shown above.

1.7 Motion tracking test

Traditional kinematic analysis characterizes the dynamics of the jet by tracking the motion of Gaussian components obtained with model fitting [e.g. 10]. With `kine`, we can move beyond this approach and estimate the continuous velocity field of the superluminal jet via video post-processing techniques. In our work we present velocity estimates using the Pyramidal Horn-Schunck optical flow algorithm [11, 12], though `kine` provides reconstructions from which this field could be easily estimated with

other algorithms. The Horn-Schunck algorithm estimates the optical flow field, which measures the pixel displacement between neighboring pairs of frames. The pixel displacement, combined with the length of the time interval between frames, gives an estimate of the jet's velocity field at each pixel location of the video. In addition, we integrate the velocity field to obtain the trajectories followed by volume elements in the jet and track significant jet components. Optical flow recovery methods are widely used in many research fields involving video observations, including astrophysics [e.g. 13].

We validated the optical flow method on `model2E`, `model2A`, `model2D`, which consist of discrete Gaussian components moving away from the core at different speeds (5.7 c, 11.4 c, 22.7 c). We computed the optical flow on the ground truth videos and the dynamic reconstructions. Then we identified the area covered by the components by thresholding the video reconstructions and for each frame and each component we averaged the velocities within the component's area to determine the component's instantaneous velocity. Results are shown in Supplementary Information Fig. 4, which shows a ground truth/reconstruction comparison for one example frame per model (rows 1-2), and the measured velocities for a few different components as a function of time, for both the ground truth and the reconstruction (rows 3-5). We report the average of the measured velocities for these components in Table 3. Again, we used the error caused by frames misalignment as uncertainty on the instantaneous velocity measurements, since it is dominant with respect to the standard deviation of the measured values. For the reconstructed videos, the measured velocities are slightly noisier than those measured for the ground truth, with noise growing with velocity, but for all models and components they are recovered correctly within 1 or 2 sigmas. We can conclude that `kine` in combination with the optical flow method, on the 3C 345 dataset, is able to accurately measure apparent velocities up to $\sim$23 c, a value above those that we measure for real observations.

	ground truth	reconstruction	true value
`model2E`			
component A	(5.7±0.2) c	(5.5±0.2) c	5.7 c
component B	(5.8±0.2) c	(5.8±0.2) c	5.7 c
component C	(5.8±0.4) c	(5.7±0.4) c	5.7 c
`model2A`			
component A	(11.1±0.2) c	(10.8±0.3) c	11.4 c
component B	(11.4±0.2) c	(11.0±0.3) c	11.4 c
component C	(11.1±0.3) c	(10.8±0.3) c	11.4 c
`model2D`			
component A	(22.1±0.4) c	(21.8±0.5) c	22.7 c
component B	(22.8±0.4) c	(22.5±0.4) c	22.7 c
component C	(22.8±0.4) c	(21.9±0.4) c	22.7 c

Table 3: **Velocity recovery test.** Apparent velocities of discrete components computed with the optical flow, for synthetic data reconstructions.

1.8 Time interpolation test

The cadence of the 3C 345 observations is irregular in time, and the separation between adjacent epochs can range from 6 days to more than 1 year. In contrast with other imaging methods, `kine` reconstructs a continuous flux density distribution, allowing for motion-preserving interpolation between observed frames, that outperforms linear interpolation. For the analysis of the fluid jet dynamics we sampled the optimized neural network at times uniformly distributed between the first and last epoch, so that the video reconstruction is not subject to errors derived from irregular sampling or frames widely separated in time. Indeed, because of the continuity of the neural field representations, a `kine` video reconstruction can be sampled at any given time, even if not coinciding with an observed epoch. A frame can be successfully interpolated if the source does not change dramatically over the interval

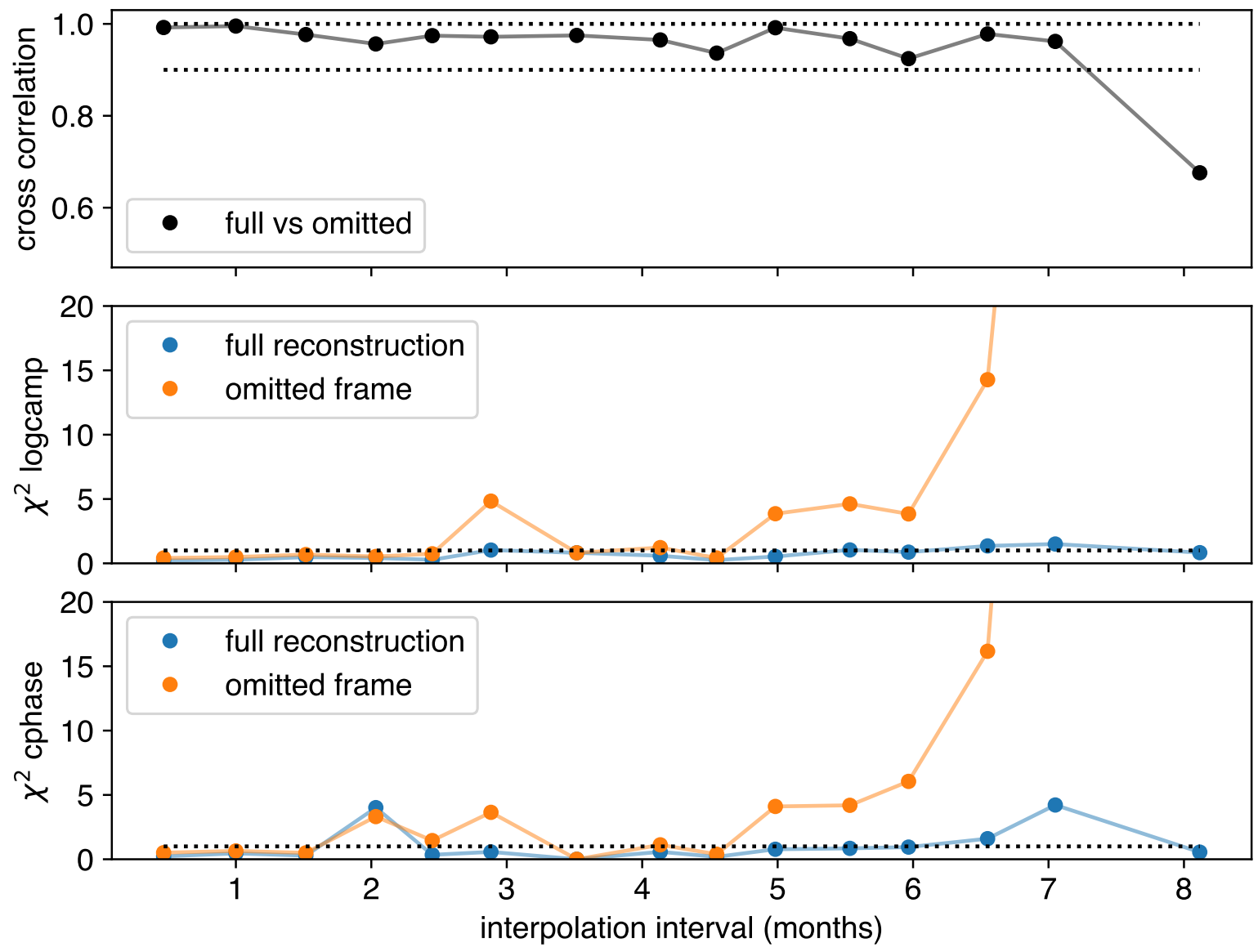

Figure 5: **Time interpolation test, metrics.** The cross correlation between the interpolated and reconstructed frames and the χ^2s of the interpolated frames are computed over time gaps of increasing length. Some example frames corresponding to the omitted epochs are compared to the corresponding frames in the full reconstruction in Supplementary Information Fig. 6.

between the previous and subsequent observation. Therefore `kine` interpolation capacity depends on both the length of the time intervals between observations and the rate of variability of the source. Here we test time interpolation in the case of 3C 345 observations. To do so, we ran `kine` reconstruction on real observations, omitting data from one observed epoch. We chose the epoch to omit so to test for increasingly longer time gaps between the omitted epoch and the nearest observation, ranging from 0.5 to 8 months. For the interpolated frame, we computed the closure quantities χ^2s to quantify if the interpolated frame provided a good fit to the data. We also computed the cross correlation between the interpolated frame and the corresponding frame from the reconstruction of the full dataset. Results are shown in Supplementary Information Fig. 5. The χ^2s shown in the plot are computed only for the selected frame, not the whole video, so, while they are expected to be of order 1, they are not expected to be all exactly 1, since the χ^2 which is minimized is the global one. Reconstructions with the omitted epoch have occasionally a slightly higher χ^2, but still not higher than 5, except in the case of a gap of 6.5 months or more. Starting from 6.5 months, the χ^2 begins to degrade. The cross correlation instead maintains high values for gaps up to 7 months, after which it decreases significantly. This is reflected in the frames presented in Supplementary Information Fig. 6.

The advantage of `kine`'s built in interpolation over more naive interpolations like linear, or bicubic interpolation, is that it locates correctly the position of moving features. For example, `kine`'s interpolation of two frames showing a moving component would show the component in the intermediate location between the initial and final point, while linear interpolation would show the presence of two dimmer components at the initial and final locations. This is exemplified in Supplementary Information Fig. 7, which compares linear interpolation and `kine`'s native interpolation for an epoch separated 6 months apart from the nearest observation, in the case of `model3A`, showing that `kine`'s interpolation is motion-preserving. In the case of real data, for the same 6 month interval, `kine`'s interpolation provides $\chi^2_{\text{logcamp}} = 3.8$ and $\chi^2_{\text{cphase}} = 6.1$, while linear interpolation results in $\chi^2_{\text{logcamp}} = 83.4$ and $\chi^2_{\text{cphase}} = 107.7$.

Overall we consider that for data with the coverage and quality of the 3C 345 observations, `kine` provides good, motion-preserving time interpolation for frames separated up to 6 months from the

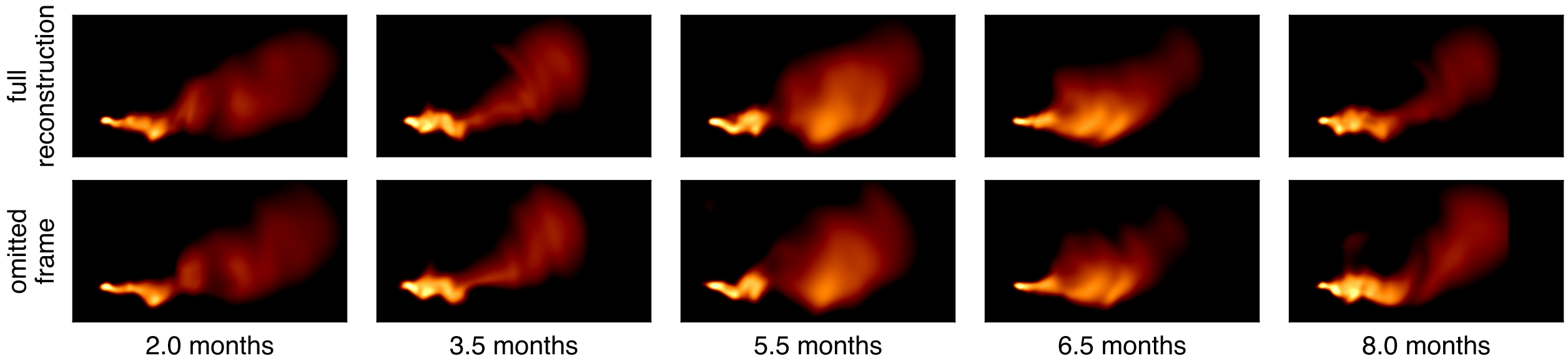

Figure 6: **Time interpolation test, frame comparison**. Reconstruction with the full set of observations (first row) and reconstruction with one omitted epoch (second row), for increasing interpolation gaps. We observe that the main features of the jet are well reconstructed in the omitted frame for all gap sizes except for the 8 months gap.

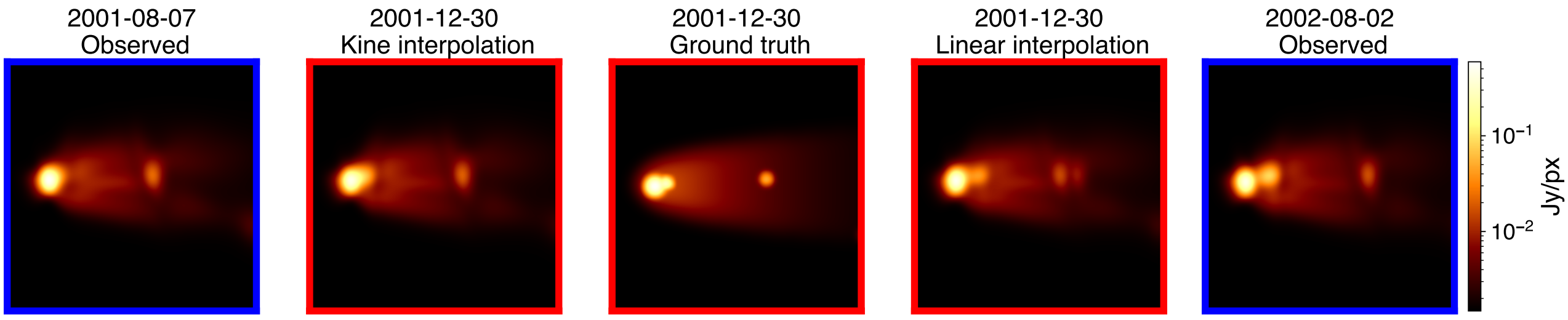

Figure 7: **Linear interpolation vs `kine`'s native interpolation.** For `model3A` and an epoch 6 months apart from the nearest observation. From left to right, the first observed frame (in blue), `kine`'s interpolated frame, the ground truth frame, and the linearly interpolated frame (in red), the second observed frame (in blue). The dynamic range shown for plotting is intentionally low, in order to highlight the position of the components against the background emission.

nearest observations. In the present work, we use time interpolated frames in the optical flow analysis to have a video sampled at regular intervals. The longest separation between an interpolated frame and the nearest observation is 5.7 months, which is within the range of reliable interpolation, therefore ensuring the reliability of the optical flow recovery.

1.9 CLEAN - kine comparison

In the dynamic imaging of 3C 345, the instantaneous coverage corresponding to one frame is the coverage of one full observation. This means that the instantaneous coverage is more than sufficient for snapshot imaging of the individual epochs, so `kine` is able to provide both dynamic and snapshot static reconstructions in super-resolution. However, as shown from the validation tests, dynamic reconstructions achieve higher resolution and dynamic range than static ones. An additional and more important advantage of a dynamic reconstruction is that a dynamic video provides a continuous representation of the movement and evolution of the source. A sequence of independent images, even if aligned correctly, always presents discontinuities from frame to frame, which cannot be overcome by traditional image interpolation. Having a continuous reconstruction is important for any image domain analysis, especially e.g. for computing the optical flow. Furthermore, the continuous formulation of `kine`'s framework allows for the sampling of the reconstructed video at any time value, providing a motion-preserving interpolation between observed frames.

Here we estimate the effective resolution and dynamic range of `kine`'s reconstruction of the real 3C 345 observations and compare them with images obtained with CLEAN. Supplementary Information Fig. 8 shows an example frame comparing traditional static imaging with CLEAN, snapshot static imaging with `kine` and dynamic imaging with `kine`. Both `kine` reconstructions show consistency with the CLEAN image, though they are able to resolve significantly more structure in the jet. The `kine` dynamic imaging reconstruction also achieves better dynamic range and resolution than both the CLEAN image and the `kine` static snapshot. A quantitative analysis of the resolution and dynamic

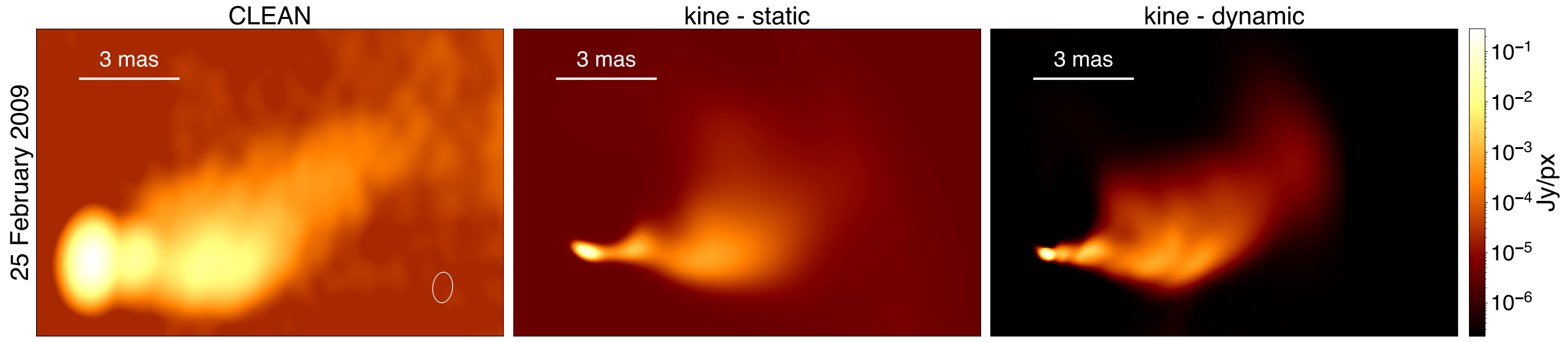

Figure 8: **CLEAN vs `kine` comparison, frames.** Comparison between representative reconstructions of VLBA observations of 3C 345 from CLEAN (left column), `kine` static (center column), and `kine` dynamic (right column). The clean image is taken from the MOJAVE database. The images have been rescaled so that the value of the brightest pixel is the same for all images and the low values are clipped to the corresponding noise level. In this way, when plotting the images with the same color scale, the difference in dynamic range is made clear by the background color of the images.

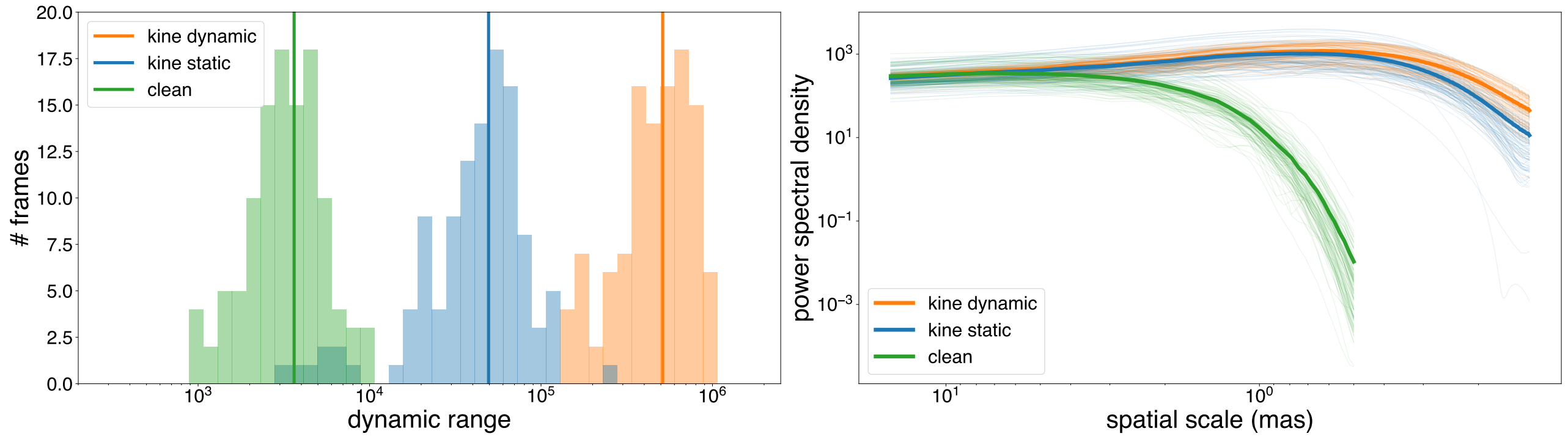

Figure 9: **CLEAN vs `kine` comparison, dynamic range and resolution.** (Left panel) Dynamic range distribution over frames of the `kine` dynamic, `kine` static, and CLEAN reconstructions of 3C 345 observations. Vertical lines indicate the average value. (Right panel) Power spectrum of the `kine` dynamic, `kine` static, and CLEAN reconstructions of 3C 345 observations. Thin lines indicate the power spectrum of each individual frame, while the thick lines indicate the average value.

range of the three reconstructions is presented in Supplementary Information Fig. 9. Since we do not have a ground truth in this case, we can estimate the dynamic range only with the traditional definition, while we estimate the resolution using the power spectrum comparison with the CLEAN model convolved with the nominal beam. The left panel of Supplementary Information Fig. 9 shows the dynamic range as a function of observed epochs, while the right panel shows the power spectrum of each frame and the average over the video.

The average dynamic range is 3.6×10^3 for CLEAN, 4.9×10^4 for `kine` static and 5.1×10^5 for `kine` dynamic, resulting in an improvement factor of ~ 14 for `kine` static over the traditional CLEAN imaging method and an additional improvement factor of ~ 10 for `kine` dynamic over `kine` static, for a total combined ~ 140 improvement factor. These values are consistent with the dynamic ranges estimated from the tests on synthetic data and with our theoretical expectations. With a 4-fold resolution improvement (discussed below), we expect up to a 16-fold dynamic range improvement for static `kine` and, with dynamic imaging of 116 epochs we expect up to a further 11-fold dynamic range improvement. The dynamic ranges of `kine` static reconstructions undergo wide oscillations, depending on the quality (coverage and SNR) of each dataset, while the dynamic range in frames of the `kine` dynamic video varies less. This means that `kine` dynamic is effective in improving the reconstruction quality of a dataset with lower data quality by leveraging the information from prior and subsequent epochs.

We obtain a resolution of 141 μas for `kine` static and a resolution of 106 μas for `kine` dynamic, representing an improvement factor of 3.4 for `kine` static and 4.5 for `kine` dynamic with respect to CLEAN, whose average nominal resolution of 475 μas is set by the diffraction limit. The effective

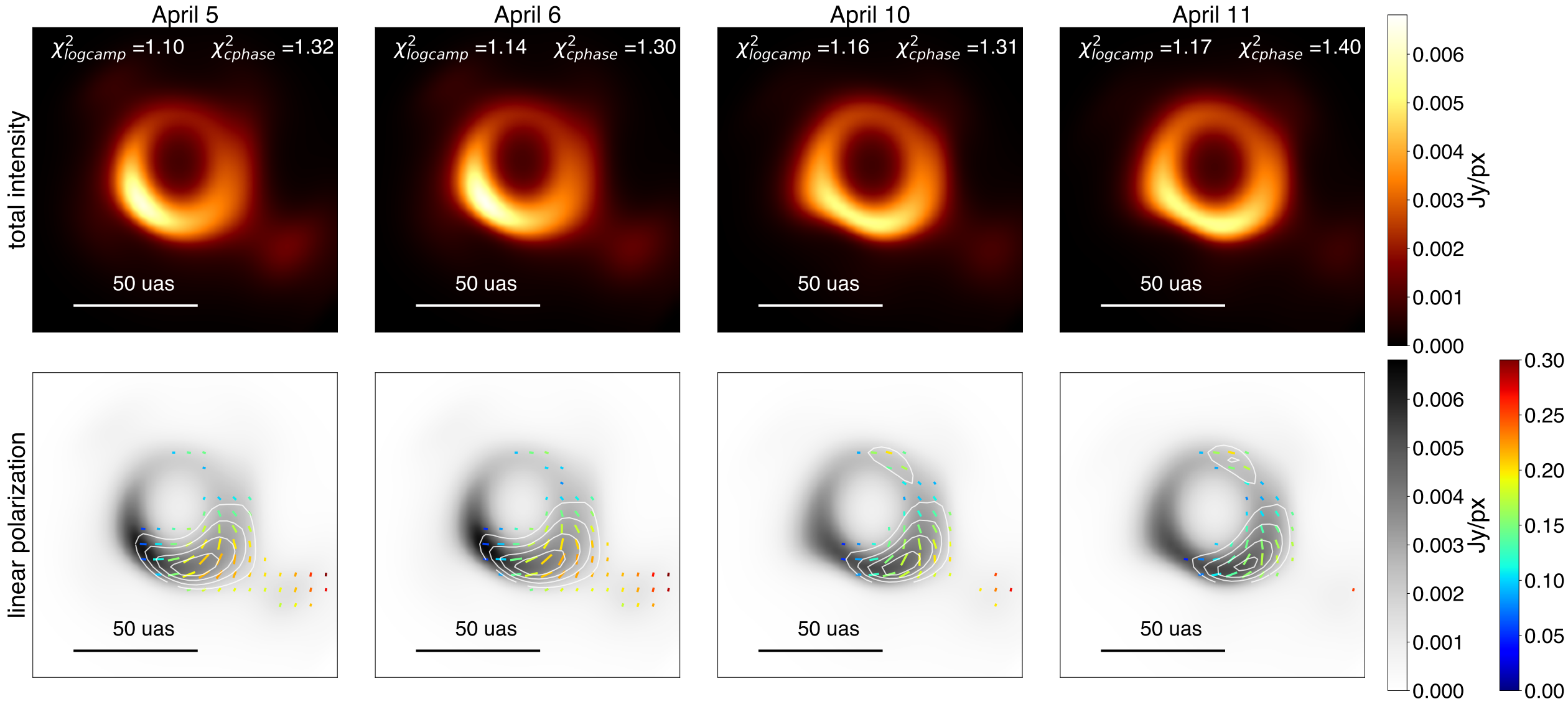

Figure 10: **M 87* `kine` reconstruction.** Multi-day dynamic imaging reconstruction of M 87*, obtained from EHT observations at 230 GHz, conducted in April 2017. The figure shows the four frames from the `kine` video that correspond to the observation days. The top panel shows the total intensity images, while the bottom panel shows the polarimetric images.

resolutions of both the static and dynamic reconstructions are consistent with the estimates from the tests on synthetic data, though we consider the resolution estimate from real data slightly less reliable than that from synthetic data because the comparison is not carried out with the ground truth but rather with the CLEAN reconstruction. Therefore we prefer to rely on the estimate from synthetic data for our conclusions.

1.10 Testing on M 87 multi-day observations

We also tested `kine` on real, multi-day observations of M 87* at 230 GHz from the EHT campaign in 2017, to validate the method on datasets with different and more sparse (u, v)-coverage. Specifically, we used observations from April 5th, 6th, 10th, and 11th of 2017 [14]. We chose this dataset because it has already been imaged by the EHT Collaboration with different static imaging methods [15], as well as by independent researchers using static imaging with CLEAN [16] and dynamic imaging with Resolve [17], making it a suitable benchmark dataset to test `kine` reconstructions.

Dynamic imaging results from `kine` are presented in Supplementary Information Fig. 10, where the frames corresponding to the four observation days are shown. The reconstructions fit the data correctly, as demonstrated by the normalized χ^2s of the closure quantities used in the imaging process. The images present a clear ring-like shape, with a brighter spot in the south-west of the ring and a twisted polarization pattern in the south-east of the ring. A faint extended emission is also visible in the south-east of the ring for all observed days, consistent with the location of the M 87 jet observed at lower frequencies [see e.g. 18, 19]. To quantify the consistency of our results with previous imaging of the dataset, we extracted the ring features from the frames at each observed day. Our results are presented in Supplementary Information Table 4 and show agreement with values from the EHT Collaboration.

	d (μas)	w (μas)	η ($^\circ$)	A	f_c
**DIFMAP**					
April 5	37.2 ± 2.4	28.2 ± 2.9	163.8 ± 6.5	0.21 ± 0.03	0.5
April 6	40.1 ± 7.4	28.6 ± 3.0	162.1 ± 9.7	0.24 ± 0.08	0.4
April 10	40.2 ± 1.7	27.5 ± 3.1	175.8 ± 9.8	0.20 ± 0.04	0.4
April 11	40.7 ± 2.6	29.0 ± 3.0	173.3 ± 4.8	0.23 ± 0.04	0.5
**eht-imaging**					
April 5	39.3 ± 1.6	16.2 ± 2.0	148.3 ± 4.8	0.25 ± 0.02	0.08
April 6	39.6 ± 1.8	16.2 ± 1.7	151.1 ± 8.6	0.25 ± 0.02	0.06
April 10	40.7 ± 1.6	15.7 ± 2.0	171.2 ± 6.9	0.23 ± 0.03	0.04
April 11	41.0 ± 1.4	15.5 ± 1.8	168.0 ± 6.9	0.20 ± 0.02	0.04
**SMILI**					
April 5	40.5 ± 1.9	16.1 ± 2.1	154.2 ± 7.1	0.27 ± 0.03	7×10^{-5}
April 6	40.9 ± 2.4	16.1 ± 2.1	151.7 ± 8.2	0.25 ± 0.02	2×10^{-4}
April 10	42.0 ± 1.8	15.7 ± 2.4	170.6 ± 5.5	0.21 ± 0.03	4×10^{-6}
April 11	42.3 ± 1.6	15.6 ± 2.2	167.6 ± 2.8	0.22 ± 0.03	6×10^{-6}
**kine**					
April 5	41.2 ± 4.0	18.2 ± 3.8	161.0 ± 21.2	0.22 ± 0.04	0.3
April 6	41.2 ± 3.6	18.0 ± 3.8	157.2 ± 18.2	0.21 ± 0.05	0.3
April 10	42.3 ± 2.7	17.3 ± 2.6	172.8 ± 29.5	0.17 ± 0.03	0.2
April 11	43.3 ± 2.9	17.4 ± 2.7	169.1 ± 33.2	0.16 ± 0.02	0.2

Table 4: **M 87* feature extraction.** Diameter d, width w, orientation angle η, asymmetry A and floor-to-ring contrast ratio f_c of the M 87* black hole shadow. `kine` results are compared with results from static imaging with DIFMAP, eht-imaging, and SMILI from the EHT imaging of M 87 [15].

References

[1] M. C. Begelman, R. D. Blandford, and M. J. Rees. Massive black hole binaries in active galactic nuclei. *Nature*, 287(5780):307–309, September 1980.

[2] A. Caproni and Z. Abraham. Precession in the inner jet of 3c 345. *Astrophys. J.*, 602(2):625–634, February 2004.

[3] A. P. Lobanov and J. Roland. A supermassive binary black hole in the quasar 3C 345. *Astron. Astrophys.*, 431(3):831–846, March 2005.

[4] Foschi, M., Gómez, J. L., Fuentes, A., Cho, I., Marscher, A. P., and Jorstad, S. Evolution, speed, and precession of the parsec-scale jet in the 3c 84 radio galaxy - super-resolved images from multi-epoch observations at 43 ghz by the very long baseline array. *A&A*, 696:A17, 2025.

[5] J. E. Pringle. Self-induced warping of accretion discs: non-linear evolution and application to agn. *Mon. Not. R. Astron. Soc.*, 292(1):136–147, nov 1997.

[6] R. J. H. Dunn, A. C. Fabian, and J. S. Sanders. Precession of the super-massive black hole in NGC 1275 (3C 84)? *Mon. Not. R. Astron. Soc.*, 366(3):758–766, 03 2006.

[7] M. Liska, C. Hesp, A. Tchekhovskoy, A. Ingram, M. van der Klis, and S. Markoff. Formation of precessing jets by tilted black hole discs in 3D general relativistic MHD simulations. *Mon. Not. R. Astron. Soc.*, 474(1):L81–L85, February 2018.

[8] M. Liska, A. Tchekhovskoy, A. Ingram, and M. van der Klis. Bardeen-Petterson alignment, jets, and magnetic truncation in GRMHD simulations of tilted thin accretion discs. *Mon. Not. R. Astron. Soc.*, 487(1):550–561, July 2019.

[9] M. Liska, C. Hesp, A. Tchekhovskoy, A. Ingram, M. van der Klis, S. B. Markoff, and M. Van Moer. Disc tearing and Bardeen-Petterson alignment in GRMHD simulations of highly tilted thin accretion discs. *Mon. Not. R. Astron. Soc.*, 507(1):983–990, October 2021.

[10] M. L. Lister, D. C. Homan, K. I. Kellermann, Y. Y. Kovalev, A. B. Pushkarev, E. Ros, and T. Savolainen. Monitoring of jets in active galactic nuclei with vlba experiments. xviii. kinematics and inner jet evolution of bright radio-loud active galaxies. *The Astrophysical Journal*, 923(1):30, dec 2021.

[11] Berthold KP Horn and Brian G Schunck. Determining optical flow. *Artificial intelligence*, 17(1-3):185–203, 1981.

[12] Enric Meinhardt-Llopis, Javier Sánchez, and Laurent Condat. Horn-schunck optical flow with a multi-scale strategy. *Image Processing On Line*, 3:151–172, 2013.

[13] Robin C. Colaninno and Angelos Vourlidas. Analysis of the velocity field of cmes using optical flow methods. *The Astrophysical Journal*, 652(2):1747, dec 2006.

[14] Event Horizon Telescope Collaboration. First m87 event horizon telescope results. ii. array and instrumentation. *The Astrophysical Journal Letters*, 875(1):L2, apr 2019.

[15] Event Horizon Telescope Collaboration. First m87 event horizon telescope results. iv. imaging the central supermassive black hole. *The Astrophysical Journal Letters*, 875(1):L4, apr 2019.

[16] Chris L. Carilli and Nithyanandan Thyagarajan. Hybrid mapping of the black hole shadow in m87. *The Astrophysical Journal*, 924(2):125, jan 2022.

[17] Philipp Arras, Philipp Frank, Philipp Haim, Jakob Knollmüller, Reimar Leike, Martin Reinecke, and Torsten Enßlin. Variable structures in m87* from space, time and frequency resolved interferometry. *Nature Astronomy*, 6:259–269, 2 2022.

[18] R. Craig Walker, Philip E. Hardee, Frederick B. Davies, Chun Ly, and William Junor. The Structure and Dynamics of the Subparsec Jet in M87 Based on 50 VLBA Observations over 17 Years at 43 GHz. *Astrophys. J.*, 855(2):128, March 2018.

[19] J. Y. Kim, T. P. Krichbaum, R. S. Lu, E. Ros, U. Bach, M. Bremer, P. de Vicente, M. Lindqvist, and J. A. Zensus. The limb-brightened jet of M87 down to the 7 Schwarzschild radii scale. *Astron. Astrophys.*, 616:A188, September 2018.
